# Algebraic evaluation of optimization in tumors classification with numerical assessments via a flask–react web interface

**DOI:** 10.3389/fdgth.2026.1767120

**Published:** 2026-06-26

**Authors:** Nouhaila Houssa, Seddik Abdelalim, Ilias Elmouki

**Affiliations:** 1 LAM2A, Faculty of Sciences Ain Chock, University Hassan II of Casablanca, Casablanca, Morocco; 2 MoNum, Hassania School of Public Works (EHTP), Casablanca, Morocco

**Keywords:** breast cancer, digital health, flask–react, health informatics, logistic regression, optimization methods, prostate cancer

## Abstract

This study addresses the practical problem of building reliable and interpretable tools to support the early detection of breast and prostate cancers. We investigate how the choice of numerical optimization method affects the training of logistic regression (LR) models for binary cancer classification. In particular, we focus on five widely used algorithms:gradient descent (GD), Newton-Raphson (NR), conjugate gradient (CG), Broyden–Fletcher–Goldfarb–Shanno (BFGS) and limited-memory BFGS (L-BFGS). We further consider ridge (L2) regularization as a standard mechanism to improve numerical stability, control coefficient magnitude, and enhance generalization reliability across datasets. Through algebraic analysis and numerical experiments on two public datasets for breast and prostate cancer, we compare these methods in terms of convergence behavior, computational efficiency, and classification performance. Our results highlight clear trade-offs between runtime, number of iterations, and predictive quality, which are directly relevant when integrating LR into real-world digital health systems. Guided by these insights and by standard practice in the literature, we implement a conventional regularized LR model in a Flask–React web application dedicated to cancer detection. In this platform, the Flask backend manages data processing, model training, and inference, while the React frontend offers an interactive interface for healthcare professionals. Clinicians can enter patient-level features and immediately visualize probabilistic predictions and class labels, illustrating how interpretable statistical models, coupled with appropriate optimization strategies, can be deployed in a web-based decision-support tool for breast and prostate cancer screening.

## 1 Introduction

Breast and prostate cancers are among the most prevalent and widely recognized malignancies worldwide, posing a significant global health burden. These cancers are major contributors to cancer mortality, with breast cancer being a leading cause of cancer death in women and prostate cancer similarly impacting men (1). High incidence and substantial mortality make early detection and timely intervention critical, since diagnosis at an earlier, more treatable stage is strongly associated with improved outcomes. For breast cancer, the U.S. Preventive Services Task Force recommends biennial mammography for women aged 40–74, recognizing that early detection through mammography can reduce mortality by facilitating early-stage diagnosis and intervention (2). For prostate cancer, cancer registry data likewise reveal substantial incidence rates and persistently high mortality, underscoring the need for early and efficient evaluation and for risk-stratified testing to identify men at higher risk of aggressive disease (3). However, no universally accepted screening guidelines exist for prostate cancer, and recommendations often vary according to age, family history and other risk factors.

Traditional diagnostic methods, including mammography, ultrasound, magnetic resonance imaging (MRI) and biopsy, remain the clinical gold standard for diagnosing breast and prostate cancers. These techniques provide invaluable information by enabling direct visualization and histopathological confirmation of suspicious lesions. Mammography is widely used to detect early-stage breast cancer by identifying suspicious masses or calcifications, while ultrasound and MRI are frequently employed to further assess abnormalities or evaluate the extent of tumor spread. Biopsy provides the definitive diagnosis by offering histopathological evidence that guides treatment decisions. Nevertheless, these tools have limitations: mammography is less sensitive in women with dense breast tissue, potentially leading to false negatives; biopsies are invasive and subject to sampling error; and imaging and pathological workflows can be time-consuming and resource-intensive.

Recent advances in machine learning (ML) have the potential to enhance the effectiveness of these traditional diagnostic methods. ML techniques can be applied to medical imaging, structured clinical data and prediction models to improve diagnostic accuracy and efficiency. For example, ML and deep learning algorithms can aid in interpreting mammograms, MRI scans and other medical images with greater precision, helping to identify subtle patterns that might be overlooked by human readers (4). ML methods can also assist in analyzing biopsy-derived or molecular data to identify signatures that better predict tumor behavior and patient prognosis (5–9). By processing large amounts of data and uncovering complex patterns, these techniques can complement traditional diagnostics, improving sensitivity, reducing false positives and refining patient risk stratification. As research in this area grows, the integration of ML into cancer diagnostics is expected to play an increasingly important role in supporting clinical decision-making.

Optimization methods play a critical role in the development and training of ML models, particularly for ensuring accurate and reliable classification outcomes. They are central to the process of estimating model parameters so that the model can learn effectively from data and converge toward an optimal solution. Various optimization algorithms, such as the Newton–Raphson method, GD, CG, (BFGS) and (L-BFGS), have been extensively studied in the context of machine learning due to their ability to efficiently estimate parameters and their well-understood convergence properties (10, 11). Each algorithm has specific strengths and weaknesses: some are particularly effective for large-scale or high-dimensional problems, whereas others may be more suitable for smaller or well-conditioned datasets. The choice of optimization technique can significantly affect convergence speed, numerical stability and, in practice, the computational resources needed to train a model.

Among ML algorithms, LR is one of the most commonly employed methods for binary classification and is widely used in medical applications such as distinguishing between benign and malignant tumors, due to its simplicity, efficiency and interpretability (12). LR models the probability that a given input belongs to a particular class, and its effectiveness depends heavily on the optimization technique used to estimate its parameters. By adjusting the optimization algorithm, it is possible to improve convergence behavior and enhance the model’s ability to make accurate predictions and generalize to unseen data.In addition, regularization techniques such as ridge (L2) regularization are commonly employed to control coefficient magnitude, improve numerical stability, and enhance the generalization ability of logistic regression models, particularly when dealing with datasets where quasi-separation or parameter instability may arise. However, LR may not always capture complex, non-linear relationships, for which alternative methods such as k-nearest neighbors (KNN) and artificial neural networks (ANNs) can be more appropriate (13, 14). These more flexible models can represent intricate patterns in medical data but often at the cost of reduced interpretability.

Deploying ML models in real-world clinical environments also requires interfaces that are functional, intuitive and user-friendly. Full-stack web applications allow healthcare professionals to interact with models and obtain real-time predictions. Frameworks such as Flask for the backend and React for the frontend are commonly used to integrate ML inference with interactive interfaces. Flask, a lightweight Python web framework, is popular for deploying ML models as web services, while React enables the creation of dynamic and responsive user interfaces tailored to clinicians’ needs (15, 16). Such platforms allow medical professionals to input patient data (e.g., clinical measurements and diagnostic features) and receive immediate, interpretable predictions from trained models, thereby supporting informed decision-making and more efficient workflows.

In this study, we focus on evaluating and comparing several optimization methods for LR models applied to breast and prostate cancer datasets. We consider models that classify tumor data as benign or malignant and examine key aspects of performance, including convergence behavior, classification accuracy and computational efficiency in terms of training time and resource use. We conduct an algebraic and numerical comparison of GD, Newton–Raphson, CG, BFGS and L-BFGS for training LR on these datasets. Based on these comparisons and standard practice in the literature, we then implement a conventional regularized LR model rather than all compared optimizers within a Flask-React web application that serves as an interactive platform for healthcare professionals. This web application enables clinicians to input relevant features, obtain probabilistic predictions and class labels, and use these outputs as a decision-support tool for breast and prostate cancer diagnosis. By linking a carefully analyzed optimization study with a practical digital health implementation, this work illustrates how optimization choices for LR can inform the design and deployment of interpretable ML tools in oncology.

### 1.1 Trustworthy, explainable, and clinically oriented medical AI

Recent medical AI research has increasingly moved beyond reporting predictive accuracy alone. Current work emphasizes transparency, reproducibility, clinical interpretability, fairness, and the practical conditions under which AI-based decision-support systems can be evaluated and deployed. Reporting guidelines such as TRIPOD+AI, CONSORT-AI, and DECIDE-AI stress the importance of clearly documenting model development, preprocessing, validation procedures, input and output handling, user interaction, and the clinical role of AI systems (17–19). These guidelines are particularly relevant for medical prediction models, since incomplete reporting can make it difficult to assess reproducibility, clinical usefulness, and potential sources of bias.

Explainable artificial intelligence has also become a central concern in healthcare. In safety-critical contexts, clinicians and researchers often need models whose decisions can be inspected and related to clinically meaningful features. Recent reviews of explainable AI in healthcare emphasize that interpretability supports trust, error analysis, and clinical adoption, especially when predictive systems are used to assist decision-making rather than operate as fully autonomous tools (20). This motivates the continued use of transparent models such as logistic regression, where coefficients can be examined directly and where regularization can improve stability without completely obscuring the decision function.

Fairness-aware learning is another important direction in trustworthy medical AI. Machine-learning models trained on biomedical datasets may inherit sampling biases, demographic imbalance, or unequal error distributions across patient groups. Reviews on fair machine learning in healthcare highlight the need to evaluate whether predictive models perform equitably across relevant subpopulations and to consider fairness throughout the machine-learning lifecycle (21). Although the present study does not perform a full fairness audit because the available datasets do not contain sufficient demographic information, the use of sensitivity, specificity, confidence intervals, and transparent model diagnostics contributes to a more clinically interpretable evaluation.

At the same time, the medical AI landscape is rapidly expanding toward more complex approaches, including AutoML systems and transformer-based healthcare models. AutoML has been proposed as a way to automate model selection and optimization in clinical applications, but recent reviews also emphasize that interpretability and rigorous validation remain essential for real-world use (22, 23). Transformer-based and large-language-model approaches have also been applied to clinical NLP, medical imaging, electronic health records, biomedical signals, and other healthcare data modalities (24). These developments show the broad methodological evolution of medical AI, but they also reinforce the need for transparent baselines and reproducible evaluation protocols.

Within this context, the present work focuses on a mathematically transparent and reproducible logistic-regression framework. The objective is not to propose a new black-box classifier, but to evaluate how different optimization strategies behave under non-regularized and ridge-regularized settings, while reporting clinically meaningful metrics, convergence diagnostics, confidence intervals, and a proof-of-concept Flask–React deployment interface. This positions the study within the current movement toward trustworthy and interpretable medical AI, while preserving a clear mathematical focus on optimization behaviour.

## 2 Mathematics of LR revisited

### 2.1 LR relevance to digital health

In many fields of medical research, especially in oncology, the classification of diseases based on various clinical features plays a crucial role in diagnosis and treatment planning. Accurate classification systems allow clinicians to distinguish between different disease states at an early stage, which is essential for improving patient outcomes and optimizing therapeutic strategies. In modern precision medicine, the integration of statistical and computational tools has significantly enhanced the ability of researchers to analyze complex biomedical data and extract meaningful patterns from clinical observations.

For instance, in breast and prostate cancer studies, researchers often aim to classify tumors as either malignant or benign based on a set of diagnostic variables, such as tumor size, shape, and patient age. Additional variables, including genetic markers, hormone receptor status, imaging features, and family medical history, are also increasingly considered to improve diagnostic accuracy. These variables collectively provide a multidimensional view of the disease and help physicians better understand tumor behavior and potential progression.

Logistic Regression (LR), a powerful statistical technique, is commonly used in such scenarios to model the relationship between a binary outcome (e.g., malignant vs. benign) and multiple explanatory variables (e.g., clinical measurements). LR is particularly valuable because it not only predicts the probability of a given outcome but also quantifies the contribution of each explanatory variable through interpretable model coefficients. This interpretability makes the method especially attractive in medical research, where understanding the influence of individual risk factors is as important as achieving high predictive performance.

This approach allows for a clear understanding of how different factors contribute to the likelihood of disease, providing valuable insights for early detection and personalized treatment. Furthermore, logistic regression models can be used to construct clinical decision-support systems that assist physicians in evaluating patient risk profiles. Such systems can guide screening programs, prioritize high-risk individuals for further diagnostic testing, and ultimately contribute to more efficient healthcare resource allocation. As a result, the use of statistical classification techniques like logistic regression plays an increasingly important role in advancing evidence-based medicine and improving the quality of patient care.

### 2.2 LR concept for breast and prostate cancer data

We aim to explain a binary variable Y that represents the state/class of the tumor being diagnosed using p explanatory variables $X_{1},\ldots ,X_{p}$ which represent, for example, in the case of the Wisconsin Breast Cancer dataset features such as: Mean radius, Mean texture, Mean perimeter, Mean area, Mean smoothness, Mean compactness, Mean concavity and Mean symmetry. For the Prostate Cancer dataset, morphological descriptors such as: Radius, Texture, Perimeter, Area, Smoothness, Compactness, Symmetry and Fractal Dim.

The data consists of n observations of $\left(Y,X_{1},\ldots ,X_{p}\right)$ denoted as:

$$\left(y_{1},x_{1,1},\ldots ,x_{1,p}),\ldots ,(y_{n},x_{n,1},\ldots ,x_{n,p}\right)$$

These observations are drawn from a population, and the set of observations forms a sample. The data is generally presented in the form of a table:

**Table:** 


Y	$X_{1}$	$X_{2}$	…	$X_{p}$
$y_{1}$	$x_{1,1}$	$x_{1,2}$	…	$x_{1,p}$
$y_{2}$	$x_{2,1}$	$x_{2,2}$	…	$x_{2,p}$
⋮	⋮	⋮	⋱	⋮
$y_{n}$	$x_{n,1}$	$x_{n,2}$	…	$x_{n,p}$

Each predictor vector is defined as:

$$x_{i,j}=(x_{i,1},x_{i,2},\ldots ,x_{i,p}{)}^{T}\in R^{\;p}$$

and the design matrix is given by:

$$X=\left[\begin{array}{ccccc} 1 & x_{1,1} & x_{1,2} & \ldots & x_{1,p}\\ 1 & x_{2,1} & x_{2,2} & \ldots & x_{2,p}\\ \vdots & \vdots & \vdots & \ddots & \vdots \\ 1 & x_{n,1} & x_{n,2} & \ldots & x_{n,p} \end{array}\right]$$

We recall that we define a (LR) and a Logit Function as follows: (LR) is a supervised learning algorithm used to model the probability of a binary outcome Y∈{0,1} given a set of predictor variables $\left(X_{1},X_{2},\ldots ,X_{p}\right)$. For each observation i∈{1,…,n}, let $\left(x_{i,1},\ldots ,x_{i,p}\right)$ be a realization of the random vector $\left(X_{1},\ldots ,X_{p}\right)$. Given $(X_{1},\ldots ,X_{p})=(x_{i,1},\ldots ,x_{i,p})=x_{i}$, the response variable $y_{i}$ is a realization of:

$$Y_{i}∼B(p(x_{i})),$$

where:

$$p(x_{i})=P(Y=1|(X_{1},\ldots ,X_{p})=x_{i}).$$

The probability of the event Y=1 is modeled using the logistic (sigmoid) function:

$$P(Y=1|(X_{1},\ldots ,X_{p}))=\sigma (X\beta )=\frac{1}{1+e^{-(\beta_{0}+\beta_{1}X_{1}+\ldots +\beta_{p}X_{p})}},=\frac{1}{1+e^{-x\prime \beta }}$$

where:

- $\sigma (z)=\frac{1}{1+e^{-z}}$ is the sigmoid function,
- $\beta =(\beta_{0},\beta_{1},\ldots ,\beta_{p})$ is the parameter vector.

The logit function is defined as the logarithm of the odds, transforming the probability function into a linear function:

$$\text{logit}(P)=\log\left(\frac{P(Y=1|(X_{1},\ldots ,X_{p}))}{1-P(Y=1|(X_{1},\ldots ,X_{p}))}\right).$$

This transformation enables the application of linear models to the log-odds of the probability.

Remark 1 The logarithm of the odds (logit function) transforms the probability function into a linear function:

$$\text{logit}(P)=\beta_{0}+\beta_{1}X_{1}+\cdots +\beta_{p}X_{p}$$ (1)

Proof. From the logistic function, we obtain the following fundamental formulations, namely (2), (3), (5), and (6):

$$P(Y=1|(X_{1},\ldots ,X_{p}))=\frac{e^{\beta_{0}+\beta_{1}X_{1}+\cdots +\beta_{p}X_{p}}}{1+e^{\beta_{0}+\beta_{1}X_{1}+\cdots +\beta_{p}X_{p}}}$$ (2)

The probability of Y=0 is:

$$P(Y=0|(X_{1},\ldots ,X_{p}))=1-P(Y=1|(X_{1},\ldots ,X_{p}))=\frac{1}{1+e^{\beta_{0}+\beta_{1}X_{1}+\cdots +\beta_{p}X_{p}}}$$ (3)

The odds of Y=1 is:

$$\frac{P(Y=1|(X_{1},\ldots ,X_{p}))}{P(Y=0|(X_{1},\ldots ,X_{p}))}=\frac{\frac{e^{\beta_{0}+\beta_{1}X_{1}+\cdots +\beta_{p}X_{p}}}{1+e^{\beta_{0}+\beta_{1}X_{1}+\cdots +\beta_{p}X_{p}}}}{\frac{1}{1+e^{\beta_{0}+\beta_{1}X_{1}+\cdots +\beta_{p}X_{p}}}}$$ (4)

Simplifying:

$$\frac{P(Y=1|(X_{1},\ldots ,X_{p}))}{P(Y=0|(X_{1},\ldots ,X_{p}))}=e^{\beta_{0}+\beta_{1}X_{1}+\cdots +\beta_{p}X_{p}}$$ (5)

Taking the logarithm:

$$\log\left(\frac{P(Y=1|(X_{1},\ldots ,X_{p}))}{1-P(Y=1|(X_{1},\ldots ,X_{p}))}\right)=\beta_{0}+\beta_{1}X_{1}+\cdots +\beta_{p}X_{p}$$ (6)

Thus, the logit function in (1) follows a linear relation with the predictor variables, proving that LR is a linear model applied to the log-odds of the probability.

Now, given $(X_{1},\ldots ,X_{p})=(x_{i,1},\ldots ,x_{i,p})=x_{i}$,let the LR function be defined as:

$$p_{\beta }(x)=P(Y=1|\left(X_{1},\ldots ,X_{p}\right))=\frac{e^{\beta_{0}+\sum_{\;j=1}^{\;p}\beta_{j}x_{j}}}{1+e^{\beta_{0}+\sum_{\;j=1}^{\;p}\beta_{j}x_{j}}}.$$

We define the mapping $p\;:\;R^{\;p+1}\to (0,1)$ as:

$$p(\beta )\;:\;x\mapsto p_{\beta }(x).$$

The model is identifiable if and only if the function p is injective, that is:

$$\forall \beta ,\beta^{\prime }\in R^{\;p+1},\;p\beta (x)=p\beta^{\prime }(x)\;\forall x\Rightarrow \beta =\beta^{\prime }.$$

Remark 2 For n>p+1, the model is identifiable if and only if the design matrix X has full rank, i.e.

rank(X)=p+1

This ensures that the predictors $x_{1},\ldots ,x_{p}$ are linearly independent, allowing for a unique estimation of the parameters β.

In fact, assume rank(X)=p+1, this means that the columns of X (that is, the variables $x_{1},\ldots ,x_{p}$) are linearly independent. If $\beta \neq \beta^{\prime }$, then $X\beta \neq X\beta^{\prime }$ (since X is full rank, hence injective). Therefore, the linear scores z=Xβ and $z^{\prime }=X\beta^{\prime }$ are different, which implies that the predicted probabilities $p_{\beta }(x)$ and $p_{\beta^{\prime }}(x)$ are different for at least one x.

Assume the model is identifiable, that is, $\beta \neq \beta^{\prime }$ implies $p_{\beta }(x)\neq p_{\beta^{\prime }}(x)$ for at least one x. If rank(X)<p+1, then the columns of X are linearly dependent. Thus, there exists a nonzero vector $\gamma \in R^{\;p+1}$ such that Xγ=0. In this case, for any β, we have X(β+γ)=Xβ. Consequently, $p_{\beta +\gamma }(x)=p_{\beta }(x)$ for all x. This contradicts the identifiability assumption, since β and β+γ are different parameters that produce the same probability distribution. Therefore, rank(X) = p+1.

Remark 3

- If X is not full rank, there is redundancy in the explanatory variables, making it impossible to uniquely estimate the parameters β.
- The condition n>p+1 ensures that X has enough rows for rank(X)=p+1 to be possible.
- If n≤p+1, the matrix X cannot be full rank (since rank(X)≤min(n,p+1)).
- In this case, the model is not identifiable, and regularization techniques (e.g., ridge or lasso regression) are required to estimate the parameters.

### 2.3 LR optimization problem

#### 2.3.1 Log-likelihood function

Recall that for the random variables $Y_{1},\ldots ,Y_{n}$ being discrete and independent, the likelihood of the LR model is defined as:

$$L_{n}\;:\;\{0,1{\}}^{n}\times R^{\;p+1}\to R$$

$$\left(y_{1},\ldots ,y_{n},\beta )\mapsto \prod_{i=1}^{n}P_{\beta }(Y_{i}=y_{i}\right)$$

where $P_{\beta }$ denotes the probability under the LR model with parameter β. For simplicity, we denote $L_{n}(y_{1},\ldots ,y_{n},\beta )=L(\beta_{0},\beta_{1},\ldots ,\beta_{p})$. The likelihood function for LR is given by:

$$L(\beta_{0},\beta_{1},\ldots ,\beta_{p})=\prod_{i=1}^{n}P(Y_{i}|X_{i}{)}^{Y_{i}}\cdot (1-P(Y_{i}|X_{i}){)}^{1-Y_{i}}$$

Remark 4 The log-likelihood function is given by:

$$\begin{array}{cc} \ell (\beta_{0},\;\beta_{1},\;\ldots ,\;\beta_{p})=\; & \sum_{i=1}^{n}[Y_{i}(\beta_{0}+\beta_{1}X_{i1}+\ldots +\beta_{p}X_{ip})\;\\ \; & -\log\left(1+e^{\beta_{0}+\beta_{1}X_{i1}+\ldots +\beta_{p}X_{ip}}\right)]\; \end{array}$$

In fact, by taking the natural logarithm of the likelihood function:

$$\ell (\beta_{0},\beta_{1},\ldots ,\beta_{p})=\log\left(\prod_{i=1}^{n}P(Y_{i}|X_{i}{)}^{Y_{i}}\cdot (1-P(Y_{i}|X_{i}){)}^{1-Y_{i}}\right)$$

This simplifies to:

$$\begin{array}{cc} \ell (\beta_{0},\;\beta_{1},\;\ldots ,\;\beta_{p})=\; & \sum_{i=1}^{n}\left[Y_{i}\log P(Y_{i}|X_{i}\right)\;\\ \; & +(1-Y_{i})\log(1-P(Y_{i}|X_{i}))]\; \end{array}$$

Substitute the logistic function $P(Y_{i}|X_{i})$ into the log-likelihood expression:

$$P(Y_{i}|X_{i})=\frac{e^{\beta_{0}+\beta_{1}X_{i1}+\ldots +\beta_{p}X_{ip}}}{1+e^{\beta_{0}+\beta_{1}X_{i1}+\ldots +\beta_{p}X_{ip}}}$$

Thus, the log-likelihood function becomes:

$$\begin{array}{cc} \ell (\beta_{0},\;\beta_{1},\;\ldots ,\;\beta_{p})=\; & \sum_{i=1}^{n}[Y_{i}\log\left(\frac{e^{\beta_{0}+\beta_{1}X_{i1}+\ldots +\beta_{p}X_{ip}}}{1+e^{\beta_{0}+\beta_{1}X_{i1}+\ldots +\beta_{p}X_{ip}}}\right)\;\\ \; & +(1-Y_{i})\log\left(\frac{1}{1+e^{\beta_{0}+\beta_{1}X_{i1}+\ldots +\beta_{p}X_{ip}}}\right)]\; \end{array}$$

Simplifying the expression, we obtain the final form of the log-likelihood function for LR:

$$\begin{array}{cc} \ell (\beta_{0},\;\beta_{1},\;\ldots ,\;\beta_{p})=\; & \sum_{i=1}^{n}[Y_{i}(\beta_{0}+\beta_{1}X_{i1}+\ldots +\beta_{p}X_{ip})\;\\ \; & -\log\left(1+e^{\beta_{0}+\beta_{1}X_{i1}+\ldots +\beta_{p}X_{ip}}\right)]\; \end{array}$$

#### 2.3.2 Estimation of parameters in LR

In LR, the goal is to estimate the parameters $\beta_{0},\beta_{1},\ldots ,\beta_{p}$ that best fit the data. The process of estimation involves maximizing the *log-likelihood function*. To achieve this, we must differentiate the log-likelihood function with respect to each parameter and solve the resulting system of equations. Recall that the maximum likelihood estimator (MLE) is defined as:

$${\hat{\beta }}_{\mathrm{MLE}}=\arg\max_{\beta \in \Theta }\ell (\beta )=\arg\min_{\beta \in \Theta }J(\beta ),$$

where J(β)=−ℓ(β) is the objective function to be minimized.

#### 2.3.3 Maximum likelihood estimation (MLE)

In the two following propositions, we need to recall that the components of a Hessian matrix H for a multivariable function

$$f(\beta_{1},\beta_{2},\ldots ,\beta_{p})$$

is defined as:

The Hessian matrix H is defined as:

$$H(f)=\left[\begin{array}{cccc} \frac{\partial^{2}f}{\partial \beta_{1}^{2}} & \frac{\partial^{2}f}{\partial \beta_{1}\partial \beta_{2}} & \cdots & \frac{\partial^{2}f}{\partial \beta_{1}\partial \beta_{p}}\\ \frac{\partial^{2}f}{\partial \beta_{2}\partial \beta_{1}} & \frac{\partial^{2}f}{\partial \beta_{2}^{2}} & \cdots & \frac{\partial^{2}f}{\partial \beta_{2}\partial \beta_{p}}\\ \vdots & \vdots & \ddots & \vdots \\ \frac{\partial^{2}f}{\partial \beta_{p}\partial \beta_{1}} & \frac{\partial^{2}f}{\partial \beta_{p}\partial \beta_{2}} & \cdots & \frac{\partial^{2}f}{\partial \beta_{p}^{2}} \end{array}\right]$$

Each element $H_{ij}$ represents the second partial derivative of f with respect to $\beta_{i}$ and $\beta_{j}$.

- The diagonal elements represent the pure second derivatives:
  $$H_{ii}=\frac{\partial^{2}f}{\partial \beta_{i}^{2}}$$
- The off-diagonal elements represent the mixed second derivatives:
  $$H_{ij}=\frac{\partial^{2}f}{\partial \beta_{i}\partial \beta_{j}}\;\text{for}\;i\neq j$$
- The Hessian matrix is symmetric if the second derivatives are continuous:
  $$H_{ij}=H_{\;ji}$$

Proposition 1 **Hessian of the log-likelihood function for two variables**

Let $\{(x_{i},y_{i}){\}}_{i=1}^{n}$ be a dataset where $x_{i}=(x_{i1},x_{i2})\in R^{2}$ and $y_{i}\in \{0,1\}$.

Consider the LR log-likelihood function:

$$\begin{array}{rl} & \ell (\beta_{0},\beta_{1},\beta_{2})=\sum_{i=1}^{n}\left[y_{i}(\beta_{0}+\beta_{1}x_{i1}+\beta_{2}x_{i2})-\log\left(1+e^{\beta_{0}+\beta_{1}x_{i1}+\beta_{2}x_{i2}}\right)\right]. \end{array}$$

Then, the Hessian matrix of ℓ with respect to $\beta =(\beta_{0},\beta_{1},\beta_{2})$ is given by:

$$H(\beta )=-\sum_{i=1}^{n}p_{i}(1-p_{i})\left(\begin{array}{ccc} 1 & x_{i1} & x_{i2}\\ x_{i1} & x_{i1}^{2} & x_{i1}x_{i2}\\ x_{i2} & x_{i1}x_{i2} & x_{i2}^{2} \end{array}\right),$$

where the estimated probability $p_{i}$ is defined by:

$$p_{i}=\frac{e^{\eta_{i}}}{1+e^{\eta_{i}}},\;\text{with}\;\eta_{i}=\beta_{0}+\beta_{1}x_{i1}+\beta_{2}x_{i2}.$$

Proof. First, we compute the first derivatives (gradient) of the log-likelihood:

$$\frac{\partial \ell }{\partial \beta_{j}}=\sum_{i=1}^{n}(y_{i}-p_{i})z_{ij},$$

where $z_{i0}=1$, $z_{i1}=x_{i1}$, and $z_{i2}=x_{i2}$.

Next, the second-order partial derivatives are:

$$\frac{\partial^{2}\ell }{\partial \beta_{j}\partial \beta_{k}}=-\sum_{i=1}^{n}p_{i}(1-p_{i})z_{ij}z_{ik}.$$

Thus, the Hessian matrix can be expressed as:

$$H(\beta )={\left[\frac{\partial^{2}\ell }{\partial \beta_{j}\partial \beta_{k}}\right]}_{\;j,k=0,1,2}=-\sum_{i=1}^{n}p_{i}(1-p_{i})\left(\begin{array}{ccc} 1 & x_{i1} & x_{i2}\\ x_{i1} & x_{i1}^{2} & x_{i1}x_{i2}\\ x_{i2} & x_{i1}x_{i2} & x_{i2}^{2} \end{array}\right).$$

This completes the proof.

Proposition 2 Let $\left(x_{i},y_{i}\right)$, i=1,…,n be a set of data points with $x_{i}\in R^{p}$ and $y_{i}\in \{0,1\}$. Suppose that the design matrix X has full rank p+1. Then, the log-likelihood function is given by:

$$\begin{array}{cc} \ell (\beta_{0},\;\beta_{1},\;\ldots ,\;\beta_{p})\; & =\sum_{i=1}^{n}[y_{i}\left(\beta_{0}+\beta_{1}X_{i1}+\cdots +\beta_{p}X_{ip}\right)\;\\ \; & -\log\left(1+\exp\left(\beta_{0}+\beta_{1}X_{i1}+\cdots +\beta_{p}X_{ip}\right)\right)]\;\\ \; & =\sum_{i=1}^{n}\left[y_{i}^{⊤}\beta -\log\left(1+e^{x_{i}^{⊤}\beta }\right)\right]\; \end{array}$$

This function is strictly concave.

Proof. To show that the log-likelihood function is strictly concave, we compute the Hessian matrix of ℓ(β). First, let’s compute the gradient of ℓ(β) with respect to the parameters $\beta =(\beta_{0},\beta_{1},\ldots ,\beta_{p})$.

The first derivative (gradient) of ℓ(β) with respect to β is:

$$\nabla_{\beta }\ell (\beta )={\left[\frac{\partial \ell (\beta )}{\partial \beta_{j}}\right]}_{0\leq j\leq p}=\sum_{i=1}^{n}\left[y_{i}x_{ij}-\frac{e^{x_{i}^{T}\beta }}{1+e^{\beta^{T}x_{i}}}x_{ij}\right]$$

Now, we compute the second derivative (Hessian matrix) of ℓ(β)

$$H(\beta )=\nabla_{\beta }^{2}\ell (\beta )={\left[\frac{\partial^{2}\ell (\beta )}{\partial \beta_{r}\partial \beta_{s}}\right]}_{0\leq r,s\leq p}=-\sum_{i=1}^{n}\left[\frac{e^{x_{i}^{T}\beta }(1+e^{x_{i}^{T}\beta })-e^{2x_{i}^{T}\beta }}{(1+e^{x_{i}^{T}\beta }{)}^{2}}x_{ir}x_{is}\right].$$

The numerator term simplified as:

$$e^{x_{i}^{T}\beta }(1+e^{x_{i}^{T}\beta })-e^{2x_{i}^{T}\beta }=e^{x_{i}^{T}\beta }\left(1+e^{x_{i}^{T}\beta }-e^{x_{i}^{T}\beta }\right)=e^{x_{i}^{T}\beta }.$$

Hence,

$$\frac{e^{x_{i}^{T}\beta }(1+e^{x_{i}^{T}\beta })-e^{2x_{i}^{T}\beta }}{(1+e^{x_{i}^{T}\beta }{)}^{2}}=\frac{e^{x_{i}^{T}\beta }}{(1+e^{x_{i}^{T}\beta }{)}^{2}}.$$

Define the sigmoid function (predicted probability):

$$p(x_{i})\;:\;=\frac{e^{x_{i}^{T}\beta }}{1+e^{x_{i}^{T}\beta }}.$$

Thus,

$$\frac{e^{x_{i}^{T}\beta }}{(1+e^{x_{i}^{T}\beta }{)}^{2}}=p(x_{i})\left(1-p(x_{i})\right).$$

Therefore, the Hessian can be expressed as:

$$H(\beta )=-\sum_{i=1}^{n}p(x_{i})(1-p(x_{i}))x_{is}x_{ir}.$$

Now, in the compact matrix form, let $X\in R^{n\times p}$ be the observation matrix or design matrix, where each row is $x_{i}^{T}$. Define the diagonal matrix $D\in R^{n\times n}$ as:

$$D=\mathrm{diag}(d_{1},\ldots ,d_{n}),\;\text{where}\;d_{i}=p(x_{i})(1-p(x_{i})).$$

Then, the Hessian can be written as:

$$H(\beta )=-X^{T}DX.$$

Let $Z\in R^{\;p+1}$ be any nonzero vector. To study concavity, we examine the quadratic form:

$$Z^{⊤}H(\beta )Z=-Z^{⊤}X^{⊤}DXZ$$

Let $D^{1/2}$ be the square root of D, which is also diagonal and positive semi-definite.

Then:

$$Z^{⊤}H(\beta )Z=0\;\Leftrightarrow \;D^{1/2}XZ=0$$

Now, since $D^{1/2}$ is invertible (as $0<p_{i}<1$ for all i), we have:

$$D^{1/2}XZ=0\;\Rightarrow \;XZ=0$$

Assume now that X is full rank: rank⁡(X)=p+1. This implies that the null space of X is trivial:

XZ=0⇒Z=0

Hence:

$$Z\neq 0\;\Rightarrow \;Z^{⊤}H(\beta )Z<0$$

Therefore, the Hessian matrix H(β) is **negative definite**, and the log-likelihood function ℓ(β) is strictly concave.

Remark 5 **Albert and Anderson, 1984**

Consider a LR model with data $\{(x_{i},y_{i}){\}}_{i=1}^{n}$, where $y_{i}\in \{0,1\}$ and $x_{i}\in R^{p}$. The maximum likelihood estimate (MLE) of β exists if and only if there does not exist a vector $\beta^{*}\in R^{p}$ such that:

$$x_{i}^{⊤}\beta^{*}>0\;\text{for all}\;i\;\text{with}\;y_{i}=1,$$

and

$$x_{i}^{⊤}\beta^{*}\leq 0\;\text{for all}\;i\;\text{with}\;y_{i}=0.$$

In other words, the MLE exists if and only if the classes are not perfectly separable by a hyperplane defined by $\beta^{*}$.

Example 1 Suppose $n_{i}=1$, and assume there exists a $\beta^{*}\in R^{p}$ such that

$$x_{i}^{⊤}\beta^{*}>0\;\text{if}\;y_{i}=1,$$

and

$$x_{i}^{⊤}\beta^{*}\leq 0\;\text{if}\;y_{i}=0.$$

The log-likelihood function is

$$l(\beta^{*})=\sum_{i=1}^{n}\left[y_{i}x_{i}^{⊤}\beta^{*}-\log\left(1+e^{x_{i}^{⊤}\beta^{*}}\right)\right].$$

We can separate the sum as

$$l(\beta^{*})=\sum_{i\;:\;y_{i}=1}\left[x_{i}^{⊤}\beta^{*}-\log\left(1+e^{x_{i}^{⊤}\beta^{*}}\right)\right]+\sum_{i\;:\;y_{i}=0}\left[-\log\left(1+e^{x_{i}^{⊤}\beta^{*}}\right)\right].\to 0$$

Consider $\alpha \beta^{*}\;\alpha >0\to +\infty$ in the direction satisfying the above inequalities:

$$l(\alpha \beta^{*})=\sum_{i\;:\;y_{i}=1}\left[\alpha x_{i}^{⊤}\beta^{*}-\log\left(1+e^{\alpha x_{i}^{⊤}\beta^{*}}\right)\right]-\sum_{i\;:\;y_{i}=0}\left[\log\left(1+e^{\alpha x_{i}^{⊤}\beta^{*}}\right)\right]\to 0$$

for $\alpha \to +\infty \;\;l(\alpha \beta^{*})\to 0$ Thus, there exists some $\beta^{*}$ such that $l(\beta^{*})\to 0$, and the MLE does not exist.

Remark 6 **The strict concavity of ℓ(β) guarantees that:**

- The log-likelihood function has a unique global maximum.
- Optimization algorithms (such as GD or Newton’s method) converge to this global maximum.

#### 2.3.4 System solution

The parameters of LR are estimated by maximizing the log-likelihood function:

$$\begin{array}{cc} \ell (\beta_{0},\;\beta_{1},\;\ldots ,\;\beta_{p})=\; & \sum_{i=1}^{n}[Y_{i}\log\left(\frac{e^{\beta_{0}+\beta_{1}X_{i}}}{1+e^{\beta_{0}+\beta_{1}X_{i}}}\right)\;\\ \; & +(1-Y_{i})\log\left(\frac{1}{1+e^{\beta_{0}+\beta_{1}X_{i}}}\right)]\; \end{array}$$ (7)

The gradient of the log-likelihood in (7) is:

$$\frac{\partial \ell (\beta_{0},\beta_{1},\ldots ,\beta_{p})}{\partial \beta_{j}}=\sum_{i=1}^{n}\left[Y_{i}-P(Y_{i}|X_{i})\right]X_{ij}=\sum_{i=1}^{n}\left[y_{i}-p_{\beta }(x_{i})\right]x_{ij}$$ (8)

Setting the gradient in (8) to zero gives the *normal equations*:

$$\frac{\partial \ell (\beta_{0},\beta_{1},\ldots ,\beta_{p})}{\partial \beta_{j}}=0\;\text{for each}\;j=0,1,\ldots ,p$$ (9)

The equations in (9) are non-linear and are generally solved using **numerical optimization methods**.

### 2.4 Computational aspects revisited

In the context of medical applications, such as classifying breast and prostate tumors as malignant or benign, LR is frequently used to model the relationship between clinical features (such as tumor size, shape, age, and genetic markers) and the binary outcome (malignant vs. benign). However, since the system of equations in LR is non-linear, it cannot be solved directly using simple algebraic methods. This is particularly true in complex medical datasets, where multiple interacting factors influence the outcome. Therefore, numerical optimization methods are typically employed to estimate the parameters of the model. These methods iteratively adjust the parameter estimates to maximize the log-likelihood function, which measures how well the model fits the observed data, enabling accurate classification of tumors in applications like early detection and treatment planning. Since the system of equations in LR is non-linear, it cannot be solved analytically. Therefore, it is generally solved using *numerical optimization methods*, which iteratively update the parameter vector to maximize the log-likelihood function (or equivalently minimize its negative).

The most common classical optimization methods include: GD,NR ,CG, BFGS, L-BFGS

#### 2.4.1 Gradient methods

##### 2.4.1.1 By using GD

The LR model (12, 25) has a log-likelihood function with gradient (score) and Hessian given by:

$$\nabla \ell (\beta )=\sum_{i=1}^{n}(y_{i}-p_{\beta }(x_{i}))x_{i},$$ (10)

$$\nabla^{2}\ell (\beta )=-\sum_{i=1}^{n}p_{\beta }(x_{i})(1-p_{\beta }(x_{i}))x_{i}x_{i}^{⊤}$$ (11)

The following Algorithm 1 summarizes the Gradient Descent optimization method used for logistic regression. The model parameters are iteratively updated in the direction of the gradient until the convergence criterion, based on the gradient norm, is satisfied.

Algorithm 1 **GD for LR (10)**

**Require:** Initial parameter $\beta^{\left(0\right)}$, step size α, tolerance ϵ

**Ensure:** Updated parameter $\beta^{\left(k+1\right)}$

 1: **for** k=0,1,2,… until convergence **do**

 2:  $\beta^{\left(k+1\right)}⇐\beta^{\left(k\right)}+\alpha \nabla \ell (\beta^{\left(k\right)})$

 3:  **if** $∥\nabla \ell (\beta^{\left(k+1\right)})∥<\epsilon$ **then**

 4:   **break**

 5:  **end if**

 6: **end for**

Note that the Hessian is negative semidefinite, hence ℓ is concave. The function is strictly concave if the design matrix has full rank and the data are not separable (26, 27).

Definition 1 **Lipschitz Gradient (L-smoothness) (28, 29)**

A differentiable function $\phi \;:\;R^{d}\to R$ has an L-Lipschitz gradient if for all u,v:

‖∇ϕ(u)−∇ϕ(v)‖≤L‖u−v‖ (12)

Equivalently, from (12) and the Descent Lemma (28), we obtain the inequality (13), for all uandv, as follows:

$$\phi (v)\leq \phi (u)+\nabla \phi (u{)}^{⊤}(v-u)+\frac{L}{2}\|v-u{\|}^{2}$$ (13)

Inspired by the Hessian bounds for the logistic loss in (30, 31) and adapting their arguments to our specific objective function ℓ(β), we obtain the following lemma.

Lemma 1 **Lipschitz Constant for Logistic Log-Likelihood**

If the covariates satisfy $\|x_{i}\|\leq R$ for all i, then the gradient of ℓ is Lipschitz with constant $L\leq \sum_{i=1}^{n}\|x_{i}{\|}^{2}/4\leq nR^{2}/4$.

From the Hessian identity as in (11), we have:

$$-\nabla^{2}\ell (\beta )=\sum_{i=1}^{n}p_{\beta }(x_{i})(1-p_{\beta }(x_{i}))x_{i}x_{i}^{⊤}$$ (14)

Since $p_{\beta }(x)(1-p_{\beta }(x))\leq 1/4$ for all real arguments and passing to the operator norm in (14), we have

$$\|\nabla^{2}\ell (\beta ){\|}_{2}\leq \sum_{i=1}^{n}p_{\beta }(x_{i})(1-p_{\beta }(x_{i}))\|x_{i}{\|}^{2}\leq \frac{1}{4}\sum_{i=1}^{n}\|x_{i}{\|}^{2}.$$ (15)

Thus, the operator norm of the Hessian, namely (15), is bounded uniformly, which implies the gradient is Lipschitz with constant $L=\underset{\beta }{\sup}\|\nabla^{2}\ell (\beta ){\|}_{2}\leq \sum_{i=1}^{n}\|x_{i}{\|}^{2}/4$.

Theorem 1 **Convergence of GD (32, 33)**

Let ℓ be the logistic log-likelihood as above. Assume covariates are bounded so that ∇ℓ is L-Lipschitz. Run GD to minimize the log-loss function J(β)=−ℓ(β):

$$\beta^{\left(k+1\right)}=\beta^{\left(k\right)}-\alpha \nabla J(\beta^{\left(k\right)})=\beta^{\left(k\right)}+\alpha \nabla \ell (\beta^{\left(k\right)}),$$ (16)

with constant step-size 0<α<2/L. Then, applying the norm in (16) leads to

$$\|\nabla \ell (\beta^{\left(k\right)})\|\to 0\text{ as }k\to \infty .$$ (17)

Proof. Let J(β)=−ℓ(β), so that ∇J(β)=−∇ℓ(β). Since ∇ℓ is L-Lipschitz, J is L-smooth. By the Descent Lemma (28), for 0<α<2/L:

$$J(\beta^{\left(k+1\right)})\leq J(\beta^{\left(k\right)})-\alpha \left(1-\frac{\alpha L}{2}\right)\|\nabla J(\beta^{\left(k\right)}){\|}^{2}.$$ (18)

Summing (18) over k=0,…,N−1 gives

$$\sum_{k=0}^{N-1}\|\nabla J(\beta^{\left(k\right)}){\|}^{2}\leq \frac{J(\beta^{\left(0\right)})-J(\beta^{\left(N\right)})}{\alpha (1-\alpha L/2)}<\infty$$ (19)

Hence, from (19) $\|\nabla J(\beta^{\left(k\right)})\|\to 0$ as k→∞, equivalently $\|\nabla \ell (\beta^{\left(k\right)})\|\to 0$.

Since

$$\nabla \ell (\beta^{\left(k\right)})=\sum_{i=1}^{n}(y_{i}-p_{\beta^{\left(k\right)}}(x_{i}))x_{i},$$ (20)

for each j. Then, for the right-hand side of (20), we have,

$$\sum_{i=1}^{n}(y_{i}-p_{\beta^{\left(k\right)}}(x_{i}))x_{ij}\to 0\;\text{as }k\to \infty .$$ (21)

Thus, from (21), we can state that the score equations are asymptotically satisfied, and under uniqueness of the MLE (34, 35) (strict concavity), $\beta^{\left(k\right)}\to {\hat{\beta }}_{\text{MLE}}$.

Theorem 2 **Linear Convergence Rate for Strongly Concave Log-Likelihood (36, 37)**

Let ℓ(β) be a μ-strongly concave and L-smooth log-likelihood function, and let α=2/(L+μ). Then, for all k≥0:

$$\ell ({\hat{\beta }}_{\text{MLE}})-\ell (\beta^{\left(k\right)})\leq {\left(\frac{L-\mu }{L+\mu }\right)}^{k}(\ell ({\hat{\beta }}_{\text{MLE}})-\ell (\beta^{\left(0\right)}))$$ (22)

where ${\hat{\beta }}_{\text{MLE}}=\arg\max_{\beta }\ell (\beta )$.

Proof. Using strong concavity (28, 38):

$$\ell ({\hat{\beta }}_{\text{MLE}})-\ell (\beta^{\left(k+1\right)})\leq \left(1-2\mu \alpha \left(1-\frac{L\alpha }{2}\right)\right)(\ell ({\hat{\beta }}_{\text{MLE}})-\ell (\beta^{\left(k\right)}))$$ (23)

Setting α=2/(L+μ) substituting this into (21) gives:

$$=\left(\frac{L-\mu }{L+\mu }\right)(\ell ({\hat{\beta }}_{\text{MLE}})-\ell (\beta^{\left(k\right)})),$$ (24)

Thus, (24) immediately gives the linear convergence rate after k iterations.

##### 2.4.1.2 By using NR Method

The NR method (10, 39, 40) is a second-order optimization algorithm that uses curvature information to achieve faster convergence.

The following Algorithm 2 summarizes the Newton–Raphson optimization procedure. At each iteration, the parameter vector is updated using the inverse Hessian matrix and the gradient of the objective function, thereby exploiting local curvature information to accelerate convergence toward a stationary point.

Algorithm 2 **NR (10)**

**Require:** Initial guess *β*^(0)^

**Ensure:** Sequence $\left(\beta^{\left(k\right)}\right)$ converging to a solution of ∇J(β)=0

 1: **for** k=0,1,2,… until convergence **do**

 2:  $\beta^{\left(k+1\right)}⇐\beta^{\left(k\right)}-[\nabla^{2}J(\beta^{\left(k\right)}){]}^{-1}\nabla J(\beta^{\left(k\right)})$

 3: **end for**

where $\nabla^{2}J(\beta^{\left(k\right)})$ is the Hessian matrix of J at $\beta^{\left(k\right)}$ (10, 41).

Theorem 3 **Quadratic Convergence (41–43)**

If $\nabla^{2}J$ is Lipschitz continuous in a neighborhood of $\beta^{*}$ and $\nabla^{2}J(\beta^{*})$ is invertible, then there exists a neighborhood of $\beta^{*}$ such that:

$$\|\beta^{\left(k+1\right)}-\beta^{*}\|\leq C\|\beta^{\left(k\right)}-\beta^{*}{\|}^{2}$$ (25)

for some constant C>0.

Proof. We proceed step by step to establish the quadratic convergence of the NR method.

**Step 1: Notations and assumptions**

Let $H(\beta )=\nabla^{2}J(\beta )$ denote the Hessian matrix of J. By the Lipschitz continuity assumption (44), there exists a neighborhood U of $\beta^{*}$ and a constant L>0 such that:

‖H(x)−H(y)‖≤L‖x−y‖,∀x,y∈U. (26)

Define the iteration error by $e_{k}=\beta^{\left(k\right)}-\beta^{*}$.

**Step 2: Taylor expansion of the gradient**

Applying the Taylor expansion (44, 45) of ∇J around $\beta^{*}$ gives:

$$\nabla J(\beta^{\left(k\right)})=\nabla J(\beta^{*})+H(\beta^{*})e_{k}+R_{k}$$ (27)

Since $\nabla J(\beta^{*})=0$, this simplifies (27) to:

$$\nabla J(\beta^{\left(k\right)})=H(\beta^{*})e_{k}+R_{k}$$ (28)

The remainder term $R_{k}$ in (28) can be written as in its integral form (45):

$$R_{k}=\int_{0}^{1}[H(\beta^{*}+te_{k})-H(\beta^{*})]e_{k}\;dt.$$ (29)

Using the Lipschitz condition (26) in (29)

$$\|R_{k}\|\leq \int_{0}^{1}\|H(\beta^{*}+te_{k})-H(\beta^{*})\|\|e_{k}\|\;dt\leq \int_{0}^{1}Lt\|e_{k}{\|}^{2}\;dt=\frac{L}{2}\|e_{k}{\|}^{2}.$$ (30)

**Step 3: Error expression after one iteration**

The Newton update rule is given by:

$$\beta^{\left(k+1\right)}=\beta^{\left(k\right)}-H(\beta^{\left(k\right)}{)}^{-1}\nabla J(\beta^{\left(k\right)}).$$ (31)

Subtracting $\beta^{*}$ in (31) yields: ?>

$$e_{k+1}=e_{k}-H(\beta^{\left(k\right)}{)}^{-1}[H(\beta^{*})e_{k}+R_{k}]$$ (32)

$$=H(\beta^{\left(k\right)}{)}^{-1}[H(\beta^{\left(k\right)})-H(\beta^{*})]e_{k}-H(\beta^{\left(k\right)}{)}^{-1}R_{k}.$$ (33)

**Step 4: Derivation of the quadratic bound**

Choose a neighborhood V⊂U small enough such that H(β) remains invertible for all β∈V, and:

$$\underset{\beta \in V}{\sup}\|H(\beta {)}^{-1}\|\leq M<\infty .$$ (34)

Applying the norm to the expression of $e_{k+1}$ from (31)–(32) and applying (30)–(34) gives: ?>

$$\left\|e_{k+1}\|\leq \|H(\beta^{\left(k\right)}{)}^{-1}\|\cdot \|H(\beta^{\left(k\right)})-H(\beta^{*})\|\cdot \|e_{k}\|+\|H(\beta^{\left(k\right)}{)}^{-1}\|\cdot \|R_{k}\right\|$$ (35)

$$\leq M\cdot L\|e_{k}{\|}^{2}+M\cdot \frac{L}{2}\|e_{k}{\|}^{2}$$ (36)

$$=\frac{3ML}{2}\|e_{k}{\|}^{2}.$$ (37)

Setting $C=\frac{3ML}{2}$, from (36)–(37), we obtain (25) as:

$$\|\beta^{\left(k+1\right)}-\beta^{*}\|\leq C\|\beta^{\left(k\right)}-\beta^{*}{\|}^{2}.$$ (38)

Hence, from (38), the NR sequence $\left\{\beta^{\left(k\right)}\right\}$ converges quadratically to $\beta^{*}$.

Theorem 4 **Local Convergence Condition (10, 29)**

Newton’s method converges locally if the Hessian matrix $\nabla^{2}J(\beta )$ is positive definite in a neighborhood of the solution.

Proof. Step 1: Descent Property Let V be a neighborhood of $\beta^{*}$ such that the Hessian matrix $H(\beta )=\nabla^{2}J(\beta )$ is positive definite for all β∈V. The Newton direction is defined as:

$$p_{N}=-H(\beta {)}^{-1}\nabla J(\beta )$$ (39)

Taking the inner product with the gradient in (39) gives:

$$\nabla J(\beta {)}^{T}p_{N}=-\nabla J(\beta {)}^{T}H(\beta {)}^{-1}\nabla J(\beta )<0$$ (40)

Note that in (40), we have ∇J(β)≠0, since $H(\beta {)}^{-1}$ is positive definite.

Step 2: Function Decrease The descent property allows us to apply a line search (46, 47) (for instance, the Armijo rule) to find $\alpha_{k}>0$ such that:

$$J(\beta^{\left(k\right)}+\alpha_{k}p_{N})<J(\beta^{\left(k\right)})$$ (41)

Step 3: Convergence The combination of the monotonic decrease of the sequence $\left\{J(\beta^{\left(k\right)})\right\}$, local compactness, and the descent property (41) ensures convergence toward a stationary point. If the initial point is sufficiently close to $\beta^{*}$, the sequence converges to $\beta^{*}$.

Moreover, if H is also Lipschitz continuous, the convergence is quadratic, according to the previous theorem.

##### 2.4.1.3 By using CG (CG) Method

The CG method (10, 48, 49) is particularly effective for large-scale optimization problems.

**Initialization:** Choose an initial value $\beta^{\left(0\right)}$ and compute the initial gradient:

$$g^{\left(0\right)}=\nabla J(\beta^{\left(0\right)})=-\nabla \ell (\beta^{\left(0\right)})$$ (42)

Note that (42) indicates the direction of steepest decrease of the objective.

The following Algorithm 3 summarizes the Conjugate Gradient (CG) optimization procedure. The method iteratively updates the parameter vector along conjugate search directions, where each direction is constructed from the current gradient and the previous direction. This approach improves convergence efficiency compared with steepest descent methods, particularly for large-scale optimization problems.

Algorithm 3 **CG (49, 50)**

**Require:** Initial parameter $\beta^{\left(0\right)}$

**Ensure:** Sequence $\left(\beta^{\left(k\right)}\right)$ approximating the minimizer of J(β)

 1: **Initial direction:** $d^{\left(0\right)}⇐-g^{\left(0\right)}=\nabla \ell (\beta^{\left(0\right)})$

 2: **for** k=0,1,2,… **do**

 3:  **Optimal step size** (47, 48):

 4:   $\alpha_{k}⇐\arg\min_{\alpha }J(\beta^{\left(k\right)}+\alpha d^{\left(k\right)})$

 5:  **Parameter update:**

 6:   $\beta^{\left(k+1\right)}⇐\beta^{\left(k\right)}+\alpha_{k}d^{\left(k\right)}$

 7:  **New gradient:**

 8:   $g^{\left(k+1\right)}⇐-\nabla \ell (\beta^{\left(k+1\right)})$

 9:  **Conjugate coefficient (Fletcher–Reeves)** :

 10:   $\beta_{k}⇐\frac{g^{(k+1)T}g^{\left(k+1\right)}}{g^{(k)T}g^{\left(k\right)}}=\frac{∥\nabla \ell (\beta^{\left(k+1\right)}){∥}^{2}}{∥\nabla \ell (\beta^{\left(k\right)}){∥}^{2}}$

 11:  **Conjugate direction:**

 12:   $d^{\left(k+1\right)}⇐-g^{\left(k+1\right)}+\beta_{k}d^{\left(k\right)}=\nabla \ell (\beta^{\left(k+1\right)})+\beta_{k}d^{\left(k\right)}$

 13:  **end for**

#### 2.4.2 Quasi-newton methods

##### 2.4.2.1 By using BFGS

Quasi-Newton methods (10) approximate the Hessian matrix to avoid expensive second-order derivative computations. The BFGS method is named after Broyden, Fletcher, Goldfarb, and Shanno (50–53).

The following Algorithm 4 summarizes the BFGS optimization procedure. At each iteration, a search direction is determined from the current inverse Hessian approximation and the gradient of the objective function. The Hessian approximation is then updated using the observed changes in the parameters and gradients, enabling superlinear convergence while avoiding the direct computation of second-order derivatives.

Algorithm 4 **BFGS (51, 52, 53, 54)**

**Require:** Initial estimate *β*^(0)^, initial approximation of the inverse Hessian $B_{0}\approx -\nabla^{2}\ell (\beta^{\left(0\right)})$

**Ensure:** Sequence (*β*^(*k*)^) approximating the maximizer of ℓ(β)

 1: **for** k=0,1,2,… **do**

 2:  **Search direction:** $d^{\left(k\right)}⇐-B_{k}^{-1}\nabla J(\beta^{\left(k\right)})=B_{k}^{-1}\nabla \ell (\beta^{\left(k\right)})$

 3:  **(since** J(β)=−ℓ(β)**)**

 4:  **Parameter update:** $\beta^{\left(k+1\right)}⇐\beta^{\left(k\right)}+\alpha_{k}d^{\left(k\right)}$

 5:  where $\alpha_{k}>0$ is obtained by a line search (48) satisfying a suitable condition

 6:  **Compute the differences:**

 7:  $s^{\left(k\right)}⇐\beta^{\left(k+1\right)}-\beta^{\left(k\right)}$

 8:  $y^{\left(k\right)}⇐\nabla J(\beta^{\left(k+1\right)})-\nabla J(\beta^{\left(k\right)})=-[\nabla \ell (\beta^{\left(k+1\right)})-\nabla \ell (\beta^{\left(k\right)})]$

 9:  **BFGS update:**

 10:  $B_{k+1}⇐B_{k}+\frac{y^{\left(k\right)}y^{(k)T}}{y^{(k)T}s^{\left(k\right)}}-\frac{B_{k}s^{\left(k\right)}s^{(k)T}B_{k}}{s^{(k)T}B_{k}s^{\left(k\right)}}$

 11: **end for**

Theorem 5 **Superlinear Convergence of BFGS (54, 55)**

Assume J is twice continuously differentiable and $\nabla^{2}J(\beta^{*})$ is positive definite. Then the BFGS method converges superlinearly:

$$\lim_{k\to \infty }\frac{\left\|\beta^{\left(k+1\right)}-\beta^{*}\right\|}{\left\|\beta^{\left(k\right)}-\beta^{*}\right\|}=0$$ (43)

Finally, (43) indicates the superlinear convergence of the BFGS method.

Proof. Before proving the theorem, we introduce the key criterion:

LEMMA 2 Dennis-Moré Criterion (56)

A quasi-Newton method converges superlinearly if and only if:

$$\lim_{k\to \infty }\frac{\left\|(B_{k}-\nabla^{2}J(\beta^{*}))s_{k}\right\|}{\left\|s_{k}\right\|}=0,$$ (44)

where $s_{k}=\beta^{\left(k+1\right)}-\beta^{\left(k\right)}$ and $B_{k}$ is the Hessian approximation.

We prove the superlinear convergence of the BFGS method in several steps.

**Step 1: BFGS Update and Secant Property**

The BFGS update is given by:

$$B_{k+1}=B_{k}+\frac{y_{k}y_{k}^{T}}{y_{k}^{T}s_{k}}-\frac{B_{k}s_{k}s_{k}^{T}B_{k}}{s_{k}^{T}B_{k}s_{k}},$$ (45)

In (45), we have

$$y_{k}=\nabla J(\beta^{\left(k+1\right)})-\nabla J(\beta^{\left(k\right)})$$ (46)

The update in (46) satisfies the secant property:

$$B_{k+1}s_{k}=y_{k}.$$ (47)

Note that (47) ensures that the updated Hessian approximation B_*k*+1_ *reproduces the observed change in the gradient along the step s*_*k*_, thereby incorporating curvature information from the most recent iteration and improving the accuracy of the Hessian approximation.

**Step 2: Express the Error Term**

Consider the difference between the Hessian approximation and the true Hessian applied to the step $s_{k}$:

$$(B_{k}-\nabla^{2}J(\beta^{*}))s_{k}=B_{k}s_{k}-\nabla^{2}J(\beta^{*})s_{k}$$ (48)

Note that the error in (48) measures how accurately *B_k_* represents the local curvature of the objective.

**Step 3: Taylor Expansion of the Gradient**

By Taylor’s theorem (44), there exists a point $\xi_{k}$ on the line segment $\left[\beta^{\left(k\right)},\beta^{\left(k+1\right)}\right]$ such that

$$y_{k}=\nabla^{2}J(\xi_{k})s_{k}.$$ (49)

Note that (49) provides a link between the secant vector and the local second-order behavior of the objective.

**Step 4: Estimate the Error Using Lipschitz Continuity**

Assuming $\nabla^{2}J$ is Lipschitz continuous with constant L:

$$\left\|y_{k}-\nabla^{2}J(\beta^{*})s_{k}\|\leq L\|\xi_{k}-\beta^{*}\|\|s_{k}\right\|$$ (50)

The bound in (50) shows that the error is proportional to both distances.

Since $\left\|\xi_{k}-\beta^{*}\|\leq \|\beta^{\left(k\right)}-\beta^{*}\right\|$ and under local superlinear convergence $\left\|\beta^{\left(k\right)}-\beta^{*}\|=o(\|s_{k}\|\right)$, it follows that

$$\left\|y_{k}-\nabla^{2}J(\beta^{*})s_{k}\|=o(\|s_{k}\|\right)$$ (51)

The quantity in (51) demonstrates that the secant vector increasingly approximates the true Hessian action near the solution.

**Step 5: Apply Dennis-Moré Criterion and Conclude**

Using the secant property and the above estimate, we have:

$$\left\|(B_{k}-\nabla^{2}J(\beta^{*}))s_{k}\|=o(\|s_{k}\|\right)$$ (52)

By the Dennis-Moré criterion (56), this proves that the BFGS method converges superlinearly. In fact, (52), together with (44), indicates that the Hessian approximation becomes increasingly accurate as the iterates approach the optimal solution.

##### 2.4.2.2 By using(L-BFGS)

For large-scale problems, the L-BFGS algorithm (57, 58) stores only a limited number of recent updates to approximate the Hessian, significantly reducing memory requirements.

The following Algorithm 5 summarizes the L-BFGS optimization procedure. At each iteration, the search direction is computed using a compact approximation of the inverse Hessian based on the most recent update pairs. The parameters are then updated through a line search, and the memory is refreshed with new curvature information, enabling efficient optimization with low storage requirements.

Algorithm 5 **L-BFGS (58, 59)**

**Require:** Initial parameter $\beta^{\left(0\right)}$, memory size m

**Ensure:** Sequence $\left(\beta^{\left(k\right)}\right)$ approximating the minimizer of J(β)

 1: Store last *m* updates $\left(s^{\left(i\right)},y^{\left(i\right)}\right)$

 2: **for** k=0,1,2,… **do**

 3:  $d^{\left(k\right)}⇐-H_{k}\nabla J(\beta^{\left(k\right)})$

 4:  $\beta^{\left(k+1\right)}⇐\beta^{\left(k\right)}+\alpha_{k}d^{\left(k\right)}$

 5:  Update memory with new pairs $\left(s^{\left(k\right)},y^{\left(k\right)}\right)$

6: **end for**

### 2.5 Regularized LR

#### 2.5.1 Ridge-regularized LR (L2)

In biomedical classification, predictors can be noisy and strongly correlated. Maximizing the unregularized log-likelihood may yield unstable coefficient estimates and poorer generalization. To improve numerical stability and control model complexity while preserving interpretability, we consider a penalized (regularized) objective using an $\ell_{2}$ (ridge) penalty. Importantly, the $\ell_{2}$ penalty keeps the objective *smooth*, so all optimization methods studied in this work (GD, NR, CG, BFGS, L-BFGS) remain directly applicable. See, e.g., (5, 29).

Definition 2 **Ridge-regularized objective**

Let ℓ(β) be the logistic regression log-likelihood and $p_{\beta }(x_{i})$ the corresponding class probability. For λ≥0, the ridge-regularized problem is defined as

$$\min_{\beta }\;J_{\lambda }(\beta )=-\ell (\beta )+\frac{\lambda }{2}\|\beta {\|}_{2}^{2}.$$ (53)

**Standing formulas (from the unregularized model).** Using the notation already introduced (Equation 10), the log-likelihood gradient (score) and Hessian are:

$$\nabla \ell (\beta )=\sum_{i=1}^{n}\left(y_{i}-p_{\beta }(x_{i})\right)x_{i},$$ (54)

$$\nabla^{2}\ell (\beta )=-\sum_{i=1}^{n}p_{\beta }(x_{i})\left(1-p_{\beta }(x_{i})\right)x_{i}x_{i}^{⊤}.$$ (55)

Proposition 3 **Gradient and Hessian of the ridge objective**

Let $J_{\lambda }(\beta )$ be defined by *(Equation 53)*. Then:

$$\nabla J_{\lambda }(\beta )=-\nabla \ell (\beta )+\lambda \beta =-\sum_{i=1}^{n}\left(y_{i}-p_{\beta }(x_{i})\right)x_{i}+\lambda \beta ,$$ (56)

and

$$\nabla^{2}J_{\lambda }(\beta )=-\nabla^{2}\ell (\beta )+\lambda I=\sum_{i=1}^{n}p_{\beta }(x_{i})\left(1-p_{\beta }(x_{i})\right)x_{i}x_{i}^{⊤}+\lambda I.$$ (57)

Proof. By linearity of differentiation, ∇(−ℓ)=−∇ℓ and $\nabla^{2}(-\ell )=-\nabla^{2}\ell$. Moreover, $\nabla \left(\frac{\lambda }{2}\|\beta {\|}_{2}^{2}\right)=\lambda \beta$ and $\nabla^{2}(\frac{\lambda }{2}\|\beta {\|}_{2}^{2})=\lambda I$. Combining these identities with (Equations 54, 55) yields (Equations 56, 57).

Proposition 4 **Convexity and uniqueness**

The objective $J_{\lambda }(\beta )$ is convex for all λ≥0. Furthermore, if λ>0, then $J_{\lambda }(\beta )$ is strictly convex and admits a unique minimizer.

Proof. For logistic regression, −ℓ(β) is convex (it has a positive semidefinite Hessian), and the ridge term $\frac{\lambda }{2}\|\beta {\|}_{2}^{2}$ is convex for λ≥0. Hence their sum is convex. When λ>0, (Equation 57) shows $\nabla^{2}J_{\lambda }(\beta )=\sum_{i}p_{\beta }(x_{i})(1-p_{\beta }(x_{i}))x_{i}x_{i}^{⊤}+\lambda I$, which is positive definite because λI≻0. Therefore, $J_{\lambda }$ is strictly convex and has a unique minimizer.

#### 2.5.2 Optimization methods

Since ridge regularization preserves smoothness, all solvers considered in the unregularized setting remain valid for minimizing $J_{\lambda }(\beta )$ by simply using the modified gradient and Hessian in Proposition 3:

- **Gradient Descent (GD):** uses $\nabla J_{\lambda }(\beta )$ in (Equation 56);
- **NR** uses both $\nabla J_{\lambda }(\beta )$ and $\nabla^{2}J_{\lambda }(\beta )$ in (Equation 57);
- **Conjugate Gradient (CG):** can be used to solve the Newton linear system without explicit matrix inversion when needed;
- **BFGS and L-BFGS:** quasi-Newton methods that use gradient information and approximate curvature.

##### 2.5.2.1 selection of λ

In the experimental section, the regularization level λ can be selected by cross-validation to ensure a fair and reproducible comparison across solvers.

## 3 Method

### 3.1 Experimental setup

In this work, we use LR as the core model for binary classification, trained with five numerical optimization methods: GD, NR, CG, BFGS and L-BFGS. For each dataset (Breast Cancer and Prostate Cancer), we compare these optimizers on convergence behaviour, runtime and predictive performance.

On the other hand, we extend our experiments to $\ell_{2}$-regularized (ridge) LR by considering a penalized formulation that discourages large coefficient magnitudes, thereby reducing overfitting and improving stability across datasets. For each dataset, the regularization strength is selected on the training set using stratified five-fold cross-validation, where the mean validation log-loss is used as the model-selection criterion; for stability, this tuning stage is carried out using L-BFGS. Using the resulting optimal regularization level, we then train the ridge-regularized model from scratch with five numerical optimization strategies (GD, NR, CG, BFGS, and L-BFGS) and compare them in terms of convergence behavior, runtime, and predictive performance (accuracy, log-loss, and ROC-AUC) on a held-out test set. We further conduct a sensitivity analysis by evaluating multiple regularization levels around the selected value, including the unregularized case.

In addition to the offline experiments comparing GD, NR, CG, BFGS and L-BFGS, we deploy a standard $\ell_{2}$-regularized LR model, as implemented in the LogisticRegression class of the scikit-learn library (59), in an interactive Flask–React web interface. In this interface, the user can manually input the tumour features through a web form and immediately obtain a binary prediction indicating whether the tumour is classified as malignant or benign.

### 3.2 Benchmark datasets

#### 3.2.1 Breast cancer wisconsin (diagnostic)

This dataset contains 569 samples with 30 real-valued features extracted from digitized breast cell nuclei images. The task is a binary classification problem: benign (0) vs malignant (1). **Basic characteristics:**

- Total samples: n=569
- Features: p=30
- Classes: 2 (Malignant = 1, Benign = 0)

**Representative features:**

- Mean radius, mean texture, mean perimeter, mean area, mean concavity, etc. (all computed from cell nuclei contours).

#### 3.2.2 Prostate cancer dataset

The Prostate Cancer dataset contains 100 samples with 8 morphological features extracted from prostate cell nuclei images. As for the previous dataset, the task is to classify tumors as benign (0) or malignant (1). **Basic characteristics:**

- Total samples: n=100
- Features: p=8
- Classes: 2 (Malignant = 1, Benign = 0)

**Feature set:**

- Radius, texture, perimeter, area, smoothness, compactness, symmetry, fractal dimension (all derived from segmented lesion regions).

### 3.3 Implementation and reproducibility details

To improve reproducibility, all experiments were performed using a fixed random seed (random_state = 42). For both datasets, the data were split into a stratified train/test partition using a 70/30 ratio, so that the class proportions were preserved in both subsets. Missing values, when present, were handled by median imputation fitted on the training set only. All numerical features were then standardized using Z-score normalization, where the StandardScaler was fitted only on the training data and subsequently applied to the test data. This procedure was used to prevent data leakage.

For clinical interpretation, the positive class was encoded as class 1. In the Breast Cancer Wisconsin dataset, the malignant class was encoded as the positive class, whereas in the Prostate Cancer dataset, the positive/cancer class was encoded as class 1. Consequently, sensitivity/recall measures the ability to detect cancer-positive cases, while specificity measures the ability to identify negative or benign cases.

All logistic-regression models were initialized at the zero vector. The intercept term was included explicitly and was not penalized by the ridge penalty. The non-regularized model was included as the special case λ=0. For the ridge-regularized model, the regularization strength was selected using stratified five-fold cross-validation on the training set by minimizing the mean validation log-loss. The candidate grid was

$$\lambda \in \{10^{-4},10^{-3},10^{-2},10^{-1},1,10,100\}.$$

The tuning step was performed with L-BFGS for numerical stability.

After selecting the optimal value of λ, the same custom logistic-regression objective, gradient, Hessian, and ridge penalty were used to compare Gradient Descent, Newton–Raphson, Conjugate Gradient, BFGS, and L-BFGS. Gradient Descent and Newton–Raphson were implemented directly, whereas Conjugate Gradient, BFGS, and L-BFGS were applied through scipy.optimize.minimize using the same custom objective and gradient functions. Therefore, the comparison focuses on optimization behaviour under a unified objective and preprocessing protocol, rather than on claiming that all numerical solvers were independently reimplemented from scratch.

The stopping criterion was based on the gradient norm. Strict convergence was declared when

$$\|\nabla J(\beta )\|<10^{-6}.$$

The maximum number of iterations was set to 8,000 for Gradient Descent, Conjugate Gradient, BFGS, and L-BFGS, and to 300 for Newton–Raphson. Predicted probabilities were converted into binary labels using the threshold 0.5. The models were evaluated on the held-out test set using accuracy, ROC-AUC, log-loss, precision, sensitivity/recall, specificity, F1-score, confusion matrices, runtime, number of iterations, and convergence status.

All experiments were implemented and executed in a Python environment on a Windows 11 workstation. The runtime environment consisted of Python 3.13.0, NumPy 2.1.2, pandas 2.2.3, SciPy 1.14.1, scikit-learn 1.5.2, and Matplotlib 3.9.2. The processor was identified as Intel64 Family 6 Model 186 Stepping 3, GenuineIntel. The experimental scripts automatically generated the reported tables and figures, thereby reducing the risk of inconsistencies between the computational outputs and the manuscript.

### 3.4 Evaluation metrics

To provide a complete assessment of predictive performance, clinical relevance, and optimization behaviour, several complementary metrics were used throughout the experiments. The predictive metrics include accuracy, ROC-AUC, and log-loss, whereas the clinical metrics include precision, sensitivity/recall, specificity, and F1-score. In addition, optimization-related quantities such as final cost, runtime, number of iterations, gradient norm, strict convergence, and optimizer success were reported in order to compare the numerical behaviour of the optimization methods. Table 1 summarizes the optimization-related quantities used to compare the numerical behaviour of the five solvers, including convergence, runtime, iteration count, and objective-function diagnostics.

**Table 1:** Optimization descriptors and convergence diagnostics used in the evaluation protocol.

Quantity	Definition	Purpose
Optimizer	GD, Newton–Raphson, CG, BFGS, or L-BFGS	Identifies the numerical method used to minimize the logistic-regression objective
λ	Ridge regularization strength	Controls the amount of $\ell_{2}$ regularization; λ=0 corresponds to the non-regularized model
Final Cost	Final value of the regularized objective J(β)	Measures the objective value reached by the optimizer
Time (s)	Total computation time in seconds	Measures computational efficiency
Iterations	Number of optimizer updates until termination	Measures convergence speed
Gradient Norm	‖∇J(β)‖	Measures proximity to a stationary point
Strict Convergence	Boolean indicator based on $\|\nabla J(\beta )\|<10^{-6}$	Indicates whether the strict convergence criterion was reached
Optimizer Success	Termination status returned by the optimization routine	Indicates whether the numerical solver reported successful termination

Table 2 reports the predictive and clinically meaningful metrics used to evaluate the classification performance of the models on the held-out test sets.

**Table 2:** Predictive and clinical evaluation metrics used in the experiments.

Metric	Definition	Purpose
Accuracy (%)	(TP+TN)/(TP+TN+FP+FN)	Measures overall classification performance
Precision	TP/(TP+FP)	Measures the reliability of positive predictions
Recall/Sensitivity	TP/(TP+FN)	Measures the ability to detect positive cancer cases
Specificity	TN/(TN+FP)	Measures the ability to correctly identify negative or benign cases
F1-score	$2\cdot \frac{\mathrm{Precision}\cdot \mathrm{Recall}}{\mathrm{Precision}+\mathrm{Recall}}$	Balances precision and recall
AUC–ROC	Area under the receiver operating characteristic curve	Measures the ranking quality of predicted probabilities
Log-loss/NLL	$-\frac{1}{n}\sum_{i}[y_{i}\log({\hat{p}}_{i})+(1-y_{i})\log(1-{\hat{p}}_{i})]$	Measures probabilistic prediction quality

Table 3 presents the diagnostic tools and uncertainty measures used to provide a more detailed interpretation of prediction errors, discrimination ability, and statistical reliability.

**Table 3:** Diagnostic tools and uncertainty measures used in the experimental analysis.

Quantity	Definition	Purpose
TP	True positives	Positive cancer cases correctly classified as positive
FP	False positives	Negative or benign cases incorrectly classified as positive
TN	True negatives	Negative or benign cases correctly classified as negative
FN	False negatives	Positive cancer cases incorrectly classified as negative
Confusion Matrix	Matrix containing TP, FP, TN, and FN	Provides detailed class-wise error analysis
ROC Curve	Plot of sensitivity against false positive rate	Visualizes discrimination performance across thresholds
Bootstrap 95% CI	Percentile interval from bootstrap resampling of test predictions	Quantifies uncertainty of the reported metrics

## 4 Results

Table 4 shows that all five optimization methods achieve the same predictive performance at the cross-validation-selected value λ=1.0, with an accuracy of 97.08%, an AUC of 0.9977, a sensitivity of 0.9375, a specificity of 0.9907, and an F1-score of 0.9600. The main differences therefore concern optimization behaviour rather than final classification performance. Newton–Raphson reaches strict convergence in only 9 iterations, whereas Gradient Descent attains comparable predictive performance but does not satisfy the strict gradient-norm criterion within the maximum number of iterations.

**Table 4:** Performance and optimization behaviour of ridge-regularized logistic regression on the Breast Cancer Wisconsin dataset.

Dataset	Optimizer	λ	Final Cost	Time (s)	Acc. (%)	AUC	Precision	Sensitivity	Specificity	F1-score	Iter.	Strict Conv.	Opt. Success
Breast cancer	GD	1.0	0.073353	1.971618	97.08	0.9977	0.9836	0.9375	0.9907	0.9600	8,000	No	No
Breast cancer	NR	1.0	0.073341	0.005959	97.08	0.9977	0.9836	0.9375	0.9907	0.9600	9	Yes	Yes
Breast cancer	CG	1.0	0.073341	0.058968	97.08	0.9977	0.9836	0.9375	0.9907	0.9600	43	Yes	Yes
Breast cancer	BFGS	1.0	0.073341	0.074615	97.08	0.9977	0.9836	0.9375	0.9907	0.9600	99	No	Yes
Breast cancer	L-BFGS	1.0	0.073341	0.015659	97.08	0.9977	0.9836	0.9375	0.9907	0.9600	29	No	Yes

Table 5 highlights the stabilizing effect of ridge regularization. Although the accuracy of Gradient Descent remains unchanged between λ=0 and λ=1.0, the log-loss decreases from 0.1003 to 0.0671, and the AUC increases from 0.9927 to 0.9977. The improvement is more pronounced for Newton–Raphson, CG, BFGS, and L-BFGS, where the non-regularized formulation yields substantially larger log-loss values. This indicates that ridge regularization improves the probabilistic quality and numerical stability of the logistic-regression framework while preserving strong clinical classification performance.

**Table 5:** Effect of ridge regularization on the Breast Cancer Wisconsin dataset. The case λ=0 corresponds to non-regularized logistic regression, while λ=1.0 is selected by stratified cross-validation using validation log-loss.

Optimizer	Setting	Accuracy (%)	AUC	Sensitivity	Specificity	F1-score	Log-loss
GD	λ=0	97.08	0.9927	0.9375	0.9907	0.9600	0.1003
GD	λ=1.0	97.08	0.9977	0.9375	0.9907	0.9600	0.0671
NR	λ=0	91.81	0.9721	0.8906	0.9346	0.8906	1.8367
NR	λ=1.0	97.08	0.9977	0.9375	0.9907	0.9600	0.0675
CG	λ=0	94.15	0.9702	0.8750	0.9813	0.9180	1.5935
CG	λ=1.0	97.08	0.9977	0.9375	0.9907	0.9600	0.0675
BFGS	λ=0	95.32	0.9581	0.9062	0.9813	0.9355	1.6159
BFGS	λ=1.0	97.08	0.9977	0.9375	0.9907	0.9600	0.0675
L-BFGS	λ=0	94.74	0.9774	0.8906	0.9813	0.9268	1.7002
L-BFGS	λ=1.0	97.08	0.9977	0.9375	0.9907	0.9600	0.0674

The bootstrap confidence intervals in Table 6 quantify the uncertainty of the final predictive and clinical metrics on the held-out test set. The intervals were computed using 2,000 non-parametric bootstrap resamples of the test predictions. Since the five optimizers yielded nearly identical predictive results at the selected regularization level, the intervals are reported for the final Newton–Raphson ridge-regularized logistic-regression model rather than for all optimizers separately.

**Table 6:** Bootstrap 95% confidence intervals for the final Newton–Raphson ridge-regularized logistic regression model on the Breast Cancer Wisconsin dataset.

Metric	Estimate	95% CI
Accuracy	0.9708	[0.9415, 0.9942]
AUC	0.9977	[0.9926, 1.0000]
Sensitivity	0.9375	[0.8710, 0.9861]
Specificity	0.9907	[0.9703, 1.0000]
Precision	0.9836	[0.9464, 1.0000]
F1-score	0.9600	[0.9174, 0.9917]
Log-loss	0.0675	[0.0345, 0.1116]

**Figure 1:**
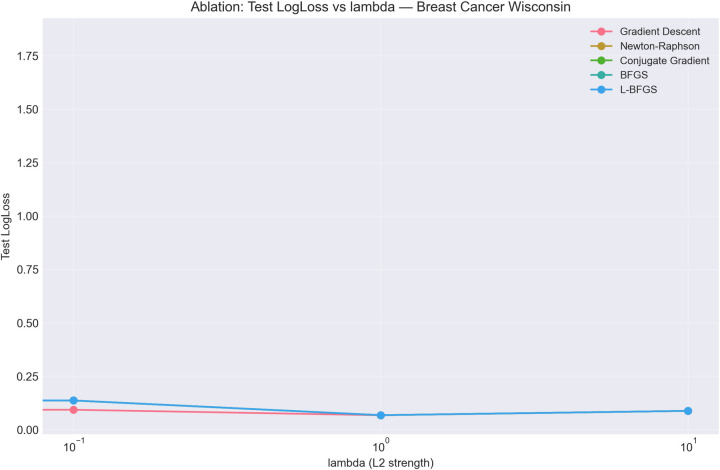
Effect of the ridge regularization parameter on the test log-loss for the Breast Cancer Wisconsin dataset. The case λ=0 corresponds to the non-regularized logistic regression model, while λ=1.0 is selected by stratified cross-validation using validation log-loss.

Figure 1 shows the effect of the ridge regularization parameter on the test log-loss for the Breast Cancer Wisconsin dataset.

**Figure 2:**
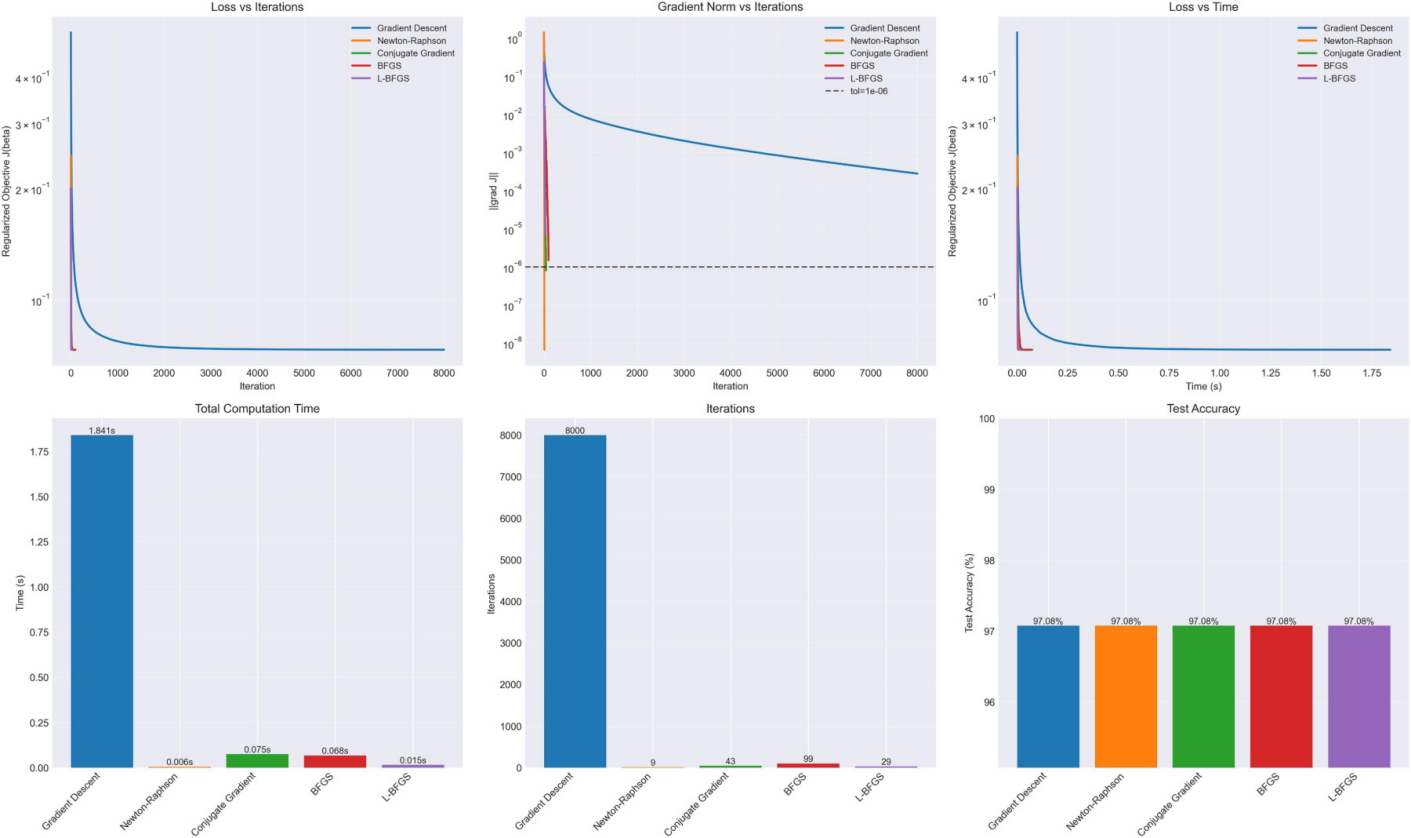
Optimization behaviour of ridge-regularized logistic regression on the Breast Cancer Wisconsin dataset at the cross-validation-selected value λ=1.0. The figure summarizes the evolution of the objective function, gradient norm, runtime, number of iterations, and test accuracy across the five optimization methods.

Figure 2 shows optimization behaviour of ridge-regularized logistic regression on the Breast Cancer Wisconsin dataset at the cross-validation-selected value λ=1.0.

**Figure 3:**
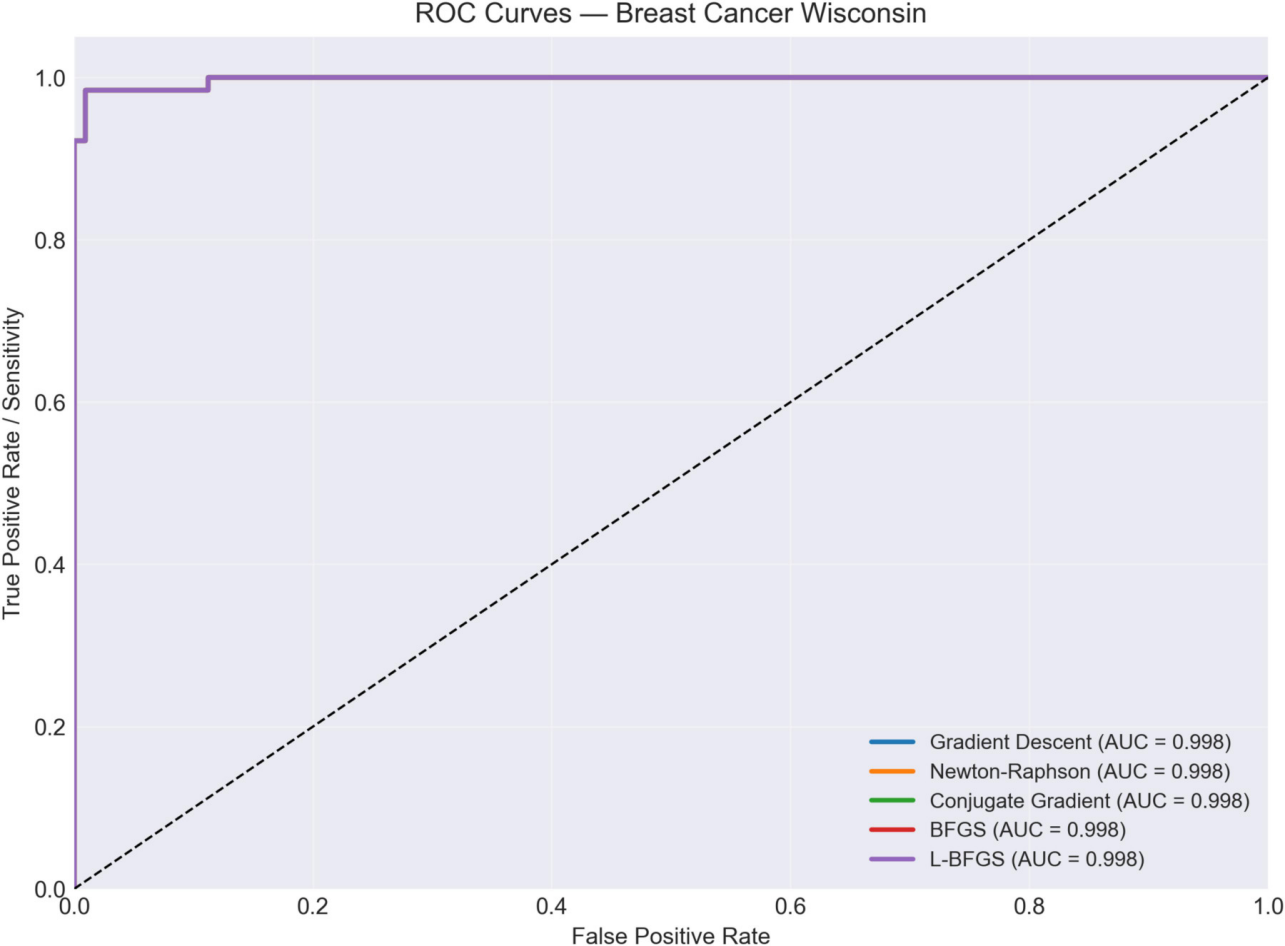
ROC curves for ridge-regularized logistic regression trained with Gradient Descent, Newton–Raphson, Conjugate Gradient, BFGS, and L-BFGS on the Breast Cancer Wisconsin dataset.

**Figure 4:**
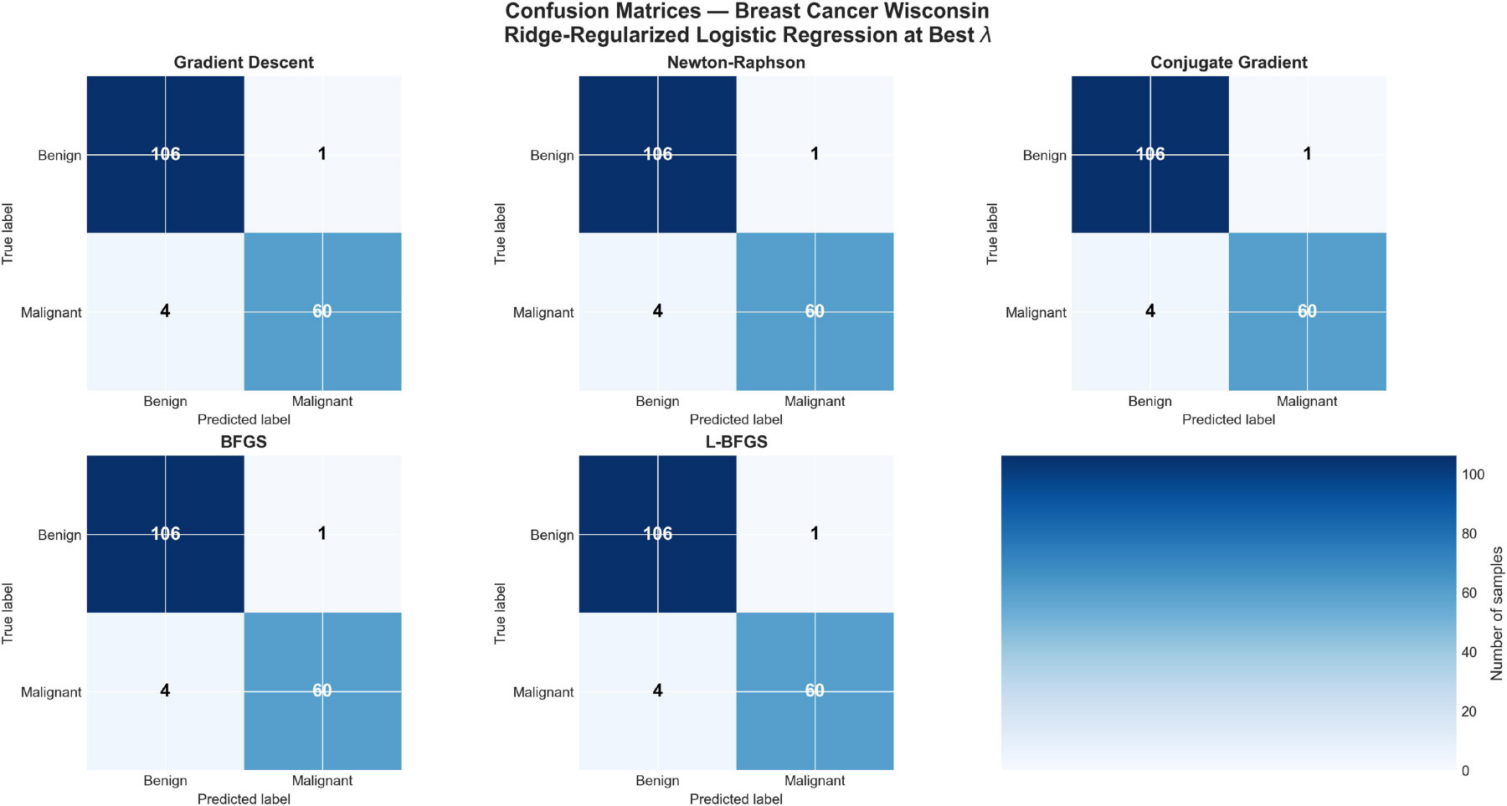
Confusion matrices obtained for the five optimization algorithms on the Breast Cancer Wisconsin dataset at the selected regularization level.

Figure 3 shows ROC curves for ridge-regularized logistic regression trained with Gradient Descent, Newton–Raphson, Conjugate Gradient, BFGS, and L-BFGS on the Breast Cancer Wisconsin dataset and Figure 4 shows Confusion matrices obtained for the five optimization algorithms.

**Figure 5:**
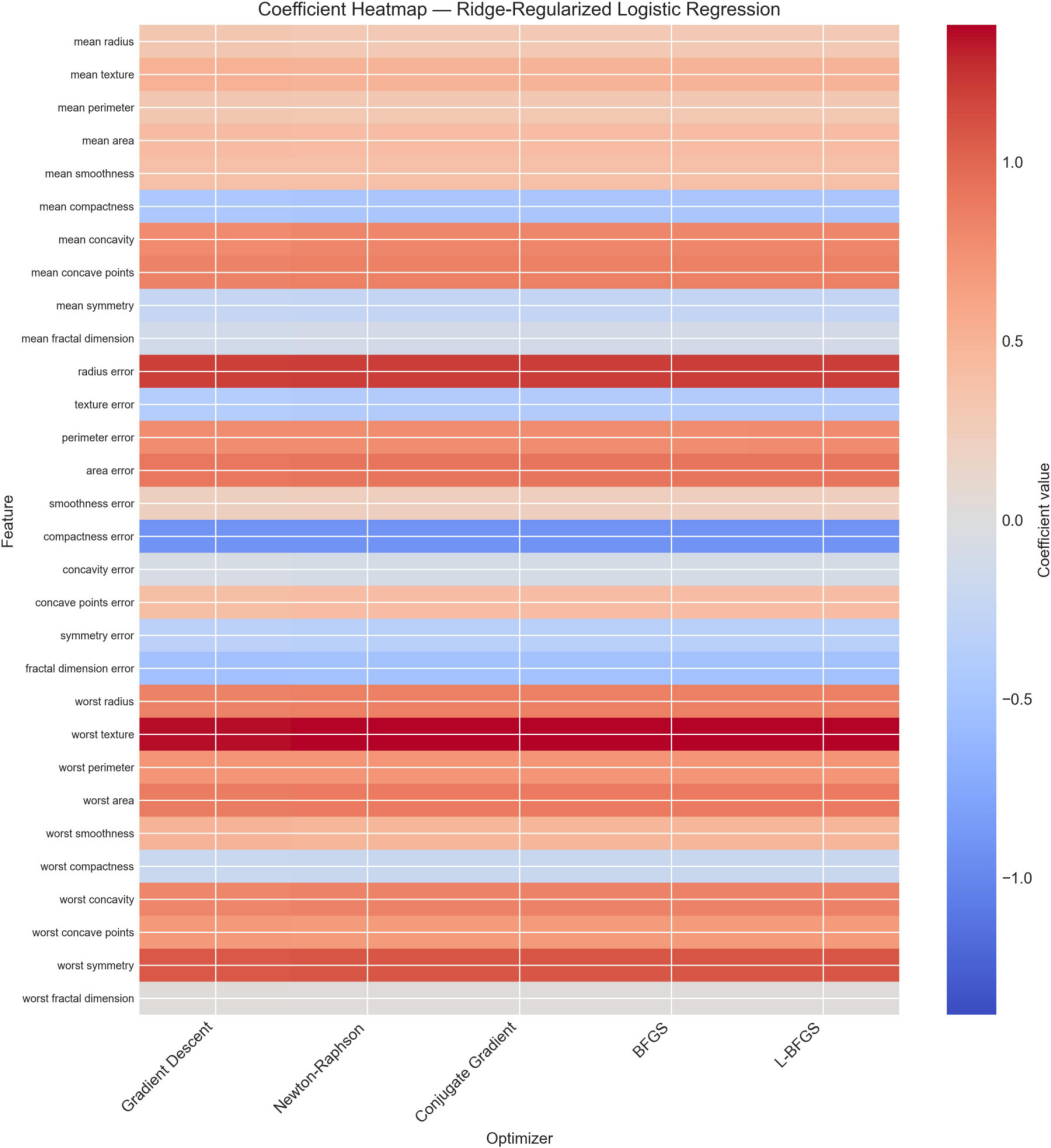
Coefficient heatmap of the ridge-regularized logistic regression model on the Breast Cancer Wisconsin dataset. The heatmap provides an interpretable view of the learned feature contributions across the different optimization methods.

Figure 5 shows Coefficient heatmap of the ridge-regularized logistic regression model on the Breast Cancer Wisconsin dataset.

Table 7 reports the final results obtained at the cross-validation-selected regularization parameter λ=10.0. All five optimization methods reach the same predictive performance, with an accuracy of 83.33%, an AUC of 0.8804, a sensitivity of 0.8947, a specificity of 0.7273, and an F1-score of 0.8718. As in the Breast Cancer experiment, the main distinction between optimizers lies in their computational behaviour rather than their final classification metrics. Newton–Raphson reaches strict convergence in only 5 iterations, while Gradient Descent requires 729 iterations.

**Table 7:** Performance and optimization behaviour of ridge-regularized logistic regression on the Prostate Cancer dataset.

Dataset	Optimizer	λ	Final Cost	Time (s)	Acc. (%)	AUC	Precision	Sensitivity	Specificity	F1-score	Iter.	Strict Conv.	Opt. Success
Prostate Cancer	GD	10.0	0.472458	0.119287	83.33	0.8804	0.8500	0.8947	0.7273	0.8718	729	Yes	Yes
Prostate Cancer	NR	10.0	0.472458	0.002078	83.33	0.8804	0.8500	0.8947	0.7273	0.8718	5	Yes	Yes
Prostate Cancer	CG	10.0	0.472458	0.009704	83.33	0.8804	0.8500	0.8947	0.7273	0.8718	11	Yes	Yes
Prostate Cancer	BFGS	10.0	0.472458	0.013043	83.33	0.8804	0.8500	0.8947	0.7273	0.8718	21	No	Yes
Prostate Cancer	L-BFGS	10.0	0.472458	0.004805	83.33	0.8804	0.8500	0.8947	0.7273	0.8718	9	No	Yes

Table 8 shows that ridge regularization improves the Prostate Cancer results across all optimizers. Compared with the non-regularized case λ=0, the cross-validation-selected value λ=10.0 increases the accuracy from 73.33% to 83.33%, improves sensitivity from 0.7895 to 0.8947, improves specificity from 0.6364 to 0.7273, and reduces the test log-loss. This supports the role of ridge regularization as a stabilizing component of the proposed logistic-regression framework.

**Table 8:** Effect of ridge regularization on the Prostate Cancer dataset. The case λ=0 corresponds to non-regularized logistic regression, while λ=10.0 is selected by stratified cross-validation using validation log-loss.

Optimizer	Setting	Accuracy (%)	AUC	Sensitivity	Specificity	F1-score	Log-loss
GD	λ=0	73.33	0.8373	0.7895	0.6364	0.7895	0.5146
GD	λ=10.0	83.33	0.8804	0.8947	0.7273	0.8718	0.4414
NR	λ=0	73.33	0.8325	0.7895	0.6364	0.7895	0.5373
NR	λ=10.0	83.33	0.8804	0.8947	0.7273	0.8718	0.4414
CG	λ=0	73.33	0.8325	0.7895	0.6364	0.7895	0.5373
CG	λ=10.0	83.33	0.8804	0.8947	0.7273	0.8718	0.4414
BFGS	λ=0	73.33	0.8325	0.7895	0.6364	0.7895	0.5373
BFGS	λ=10.0	83.33	0.8804	0.8947	0.7273	0.8718	0.4414
L-BFGS	λ=0	73.33	0.8325	0.7895	0.6364	0.7895	0.5373
L-BFGS	λ=10.0	83.33	0.8804	0.8947	0.7273	0.8718	0.4414

The bootstrap confidence intervals in Table 9 quantify the uncertainty of the final predictive and clinical metrics on the held-out test set. The intervals were computed using 2,000 non-parametric bootstrap resamples of the test predictions. Since the five optimizers yielded identical predictive results at the selected regularization level, the intervals are reported for the final Newton–Raphson ridge-regularized logistic-regression model rather than for all optimizers separately.

**Table 9:** Bootstrap 95% confidence intervals for the final Newton–Raphson ridge-regularized logistic regression model on the Prostate Cancer dataset.

Metric	Estimate	95% CI
Accuracy	0.8333	[0.7000, 0.9667]
AUC	0.8804	[0.7159, 0.9861]
Sensitivity	0.8947	[0.7368, 1.0000]
Specificity	0.7273	[0.4286, 1.0000]
Precision	0.8500	[0.6818, 1.0000]
F1-score	0.8718	[0.7429, 0.9730]
Log-loss	0.4414	[0.3391, 0.5510]

**Figure 6:**
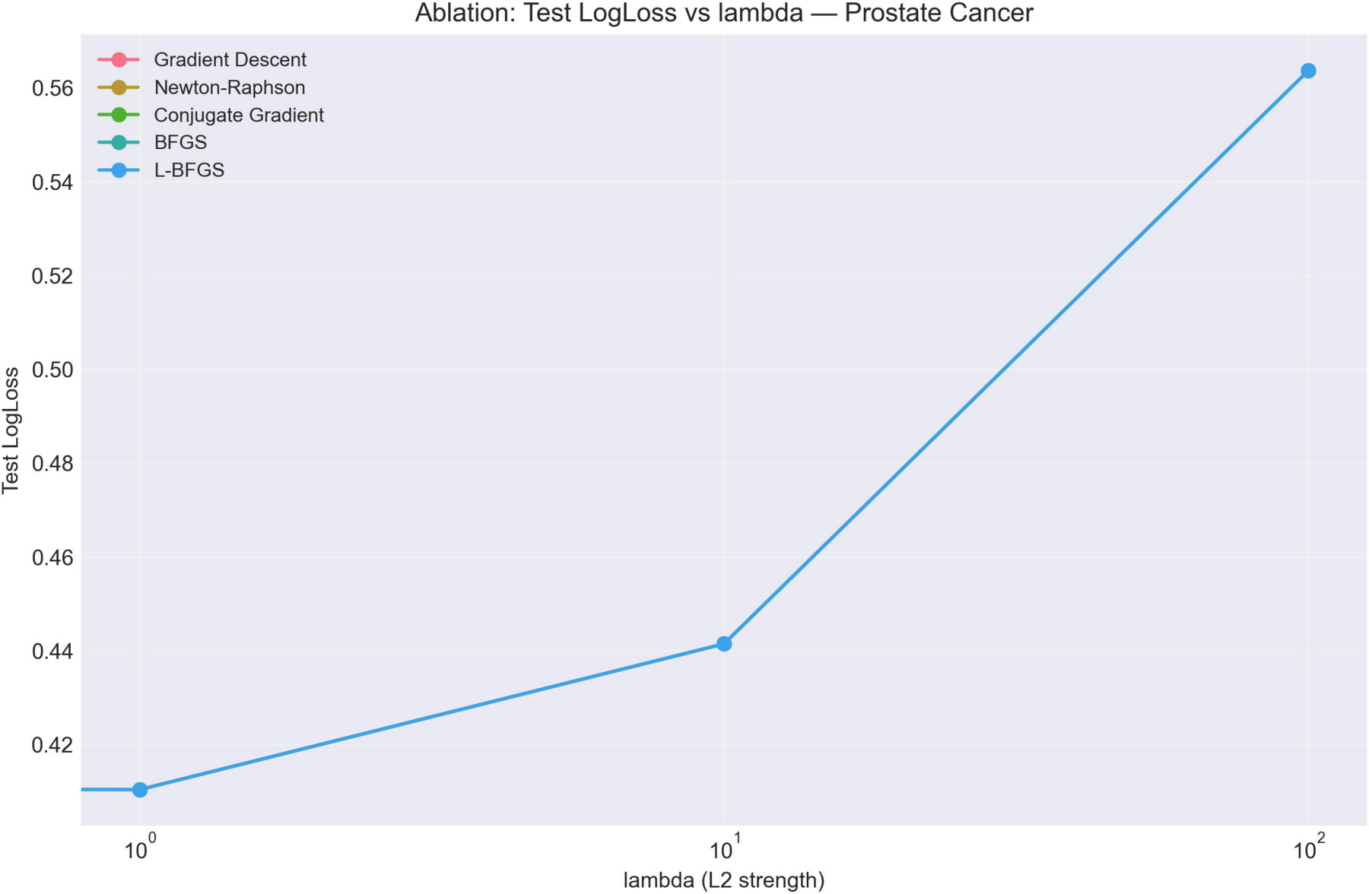
Effect of the ridge regularization parameter on the test log-loss for the Prostate Cancer dataset. The case λ=0 corresponds to the non-regularized logistic regression model, while λ=10.0 is selected by stratified cross-validation using validation log-loss.

Figure 6 shows the effect of the ridge regularization parameter on the test log-loss forthe Prostate Cancer dataset.

**Figure 7:**
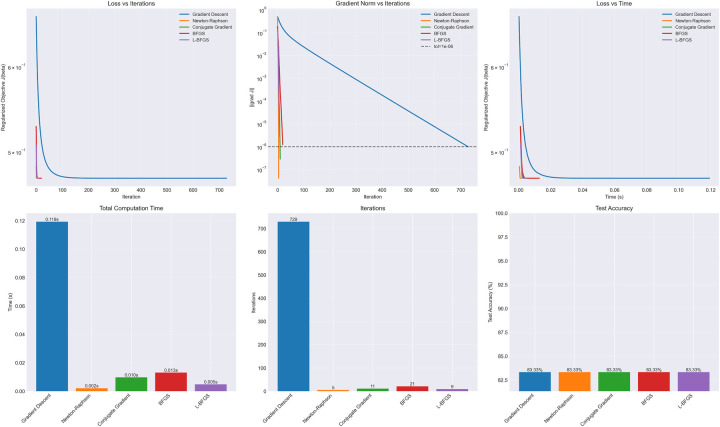
Optimization behaviour of ridge-regularized logistic regression on the Prostate Cancer dataset at the cross-validation-selected value λ=10.0. The figure summarizes the evolution of the objective function, gradient norm, runtime, number of iterations, and test accuracy across the five optimization methods.

Figure 7 shows optimization behaviour of ridge-regularized logistic regression on the Prostate Cancer dataset at the cross-validation-selected value λ=10.0.

**Figure 8:**
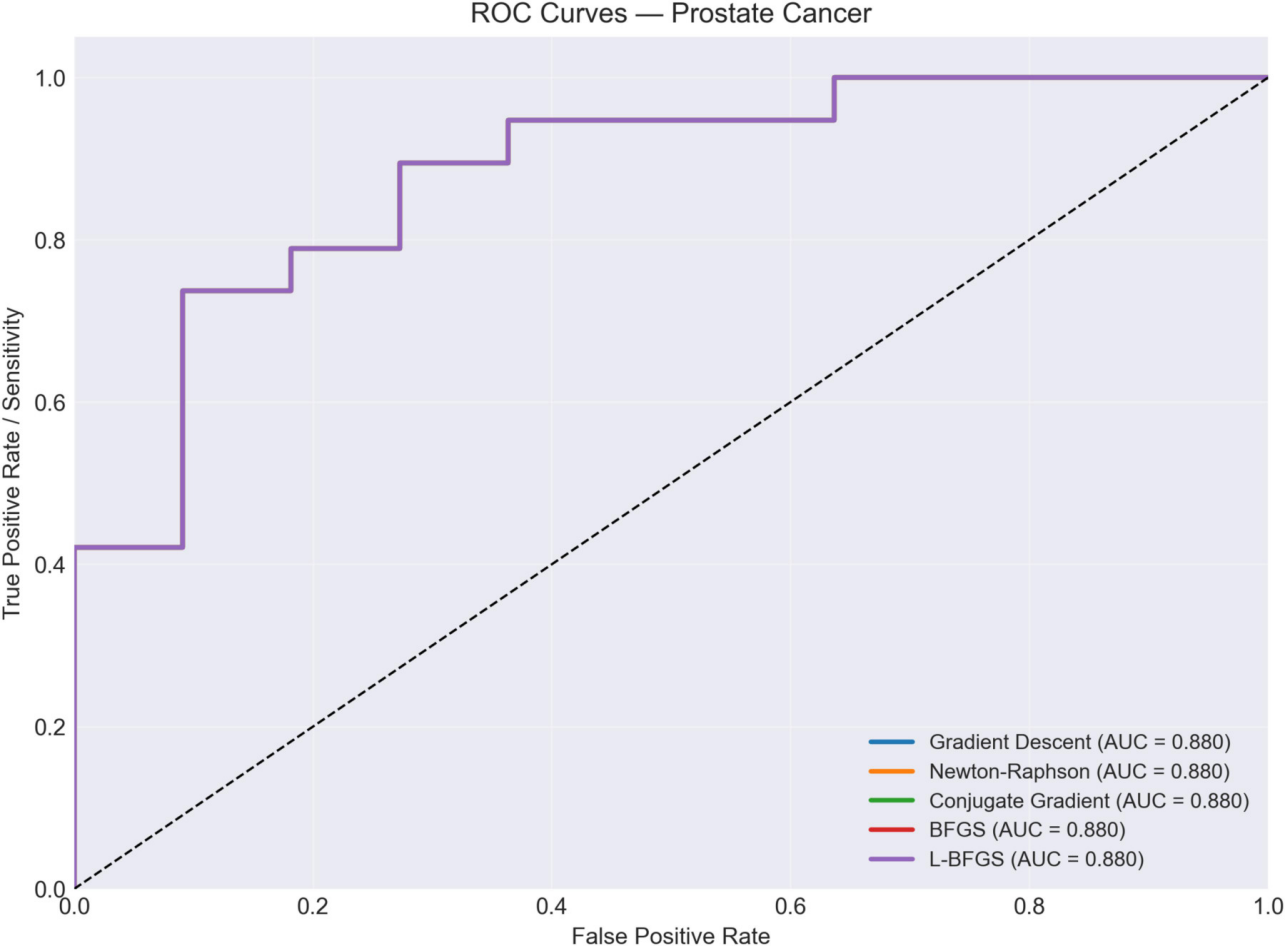
ROC curves for ridge-regularized logistic regression trained with Gradient Descent, Newton–Raphson, Conjugate Gradient, BFGS, and L-BFGS on the Prostate Cancer dataset.

**Figure 9:**
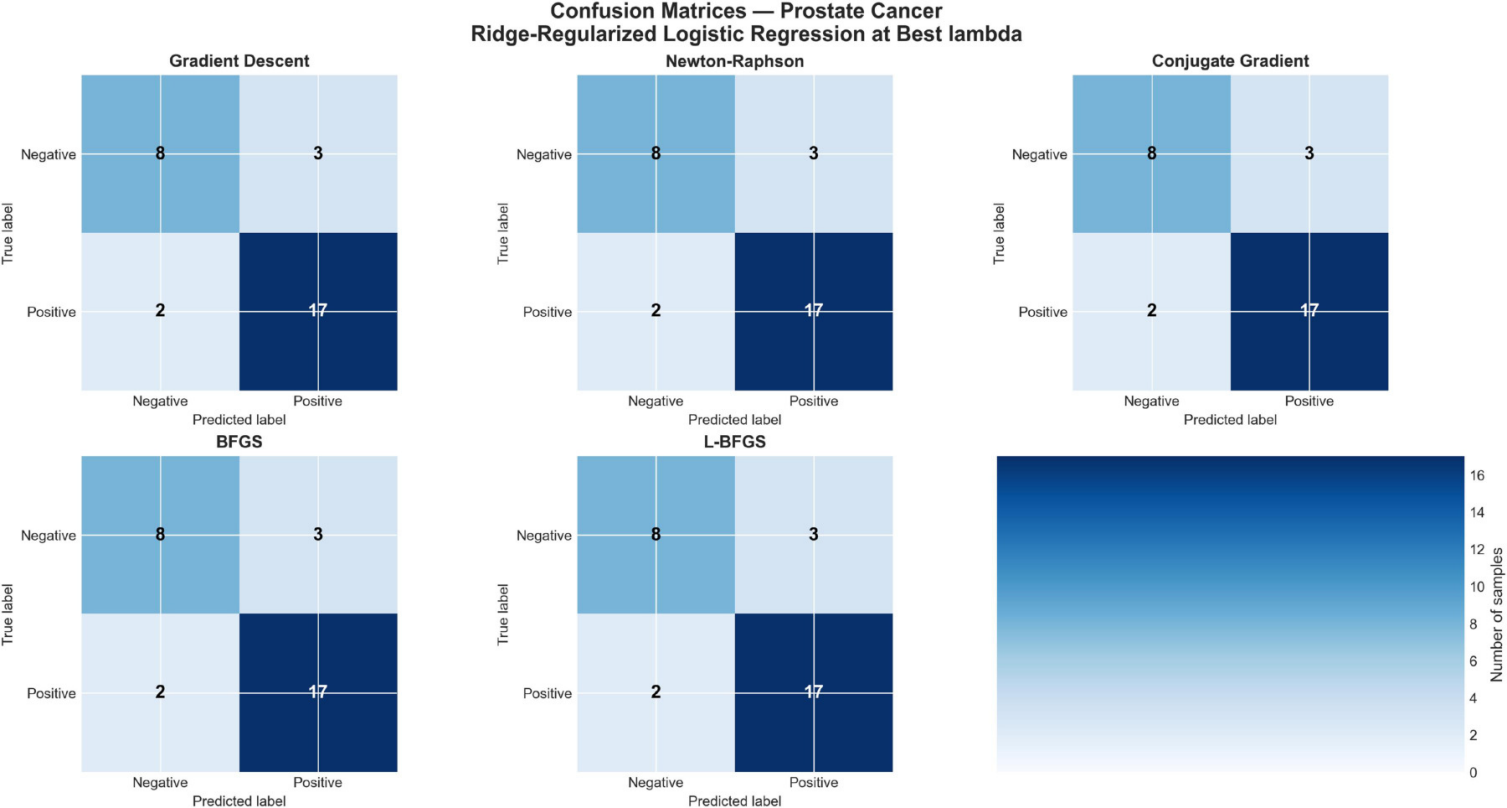
Confusion matrices obtained for the five optimization algorithms on the Prostate Cancer dataset at the selected regularization level. The positive/cancer class is treated as the positive class; therefore, sensitivity corresponds to cancer-case detection and specificity corresponds to negative-case identification.

Figure 8 shows ROC curves for ridge-regularized logistic regression trained with Gradient Descent, Newton–Raphson, Conjugate Gradient, BFGS, and L-BFGS on on the Prostate Cancer dataset. and Figure 9 shows Confusion matrices obtained for the five optimization algorithms.

**Figure 10:**
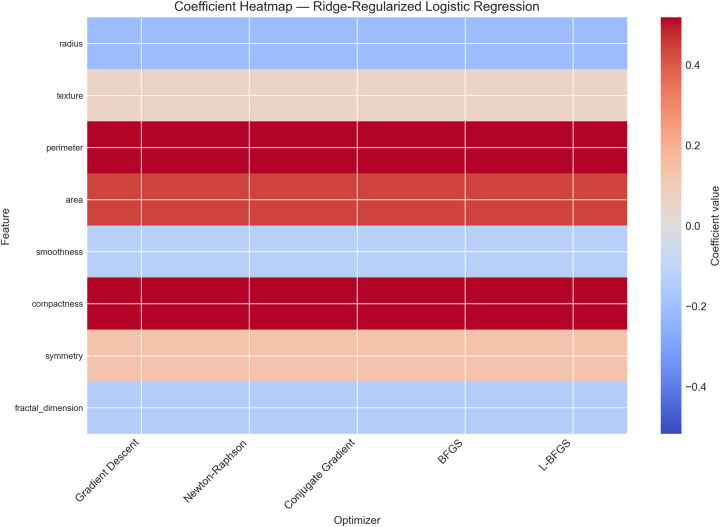
Coefficient heatmap of the ridge-regularized logistic regression model on the Prostate Cancer dataset. The heatmap provides an interpretable view of the standardized feature contributions across the different optimization methods.

Figure 10 shows Coefficient heatmap of the ridge-regularized logistic regression model on the Prostate Cancer dataset.

**Figure 11:**
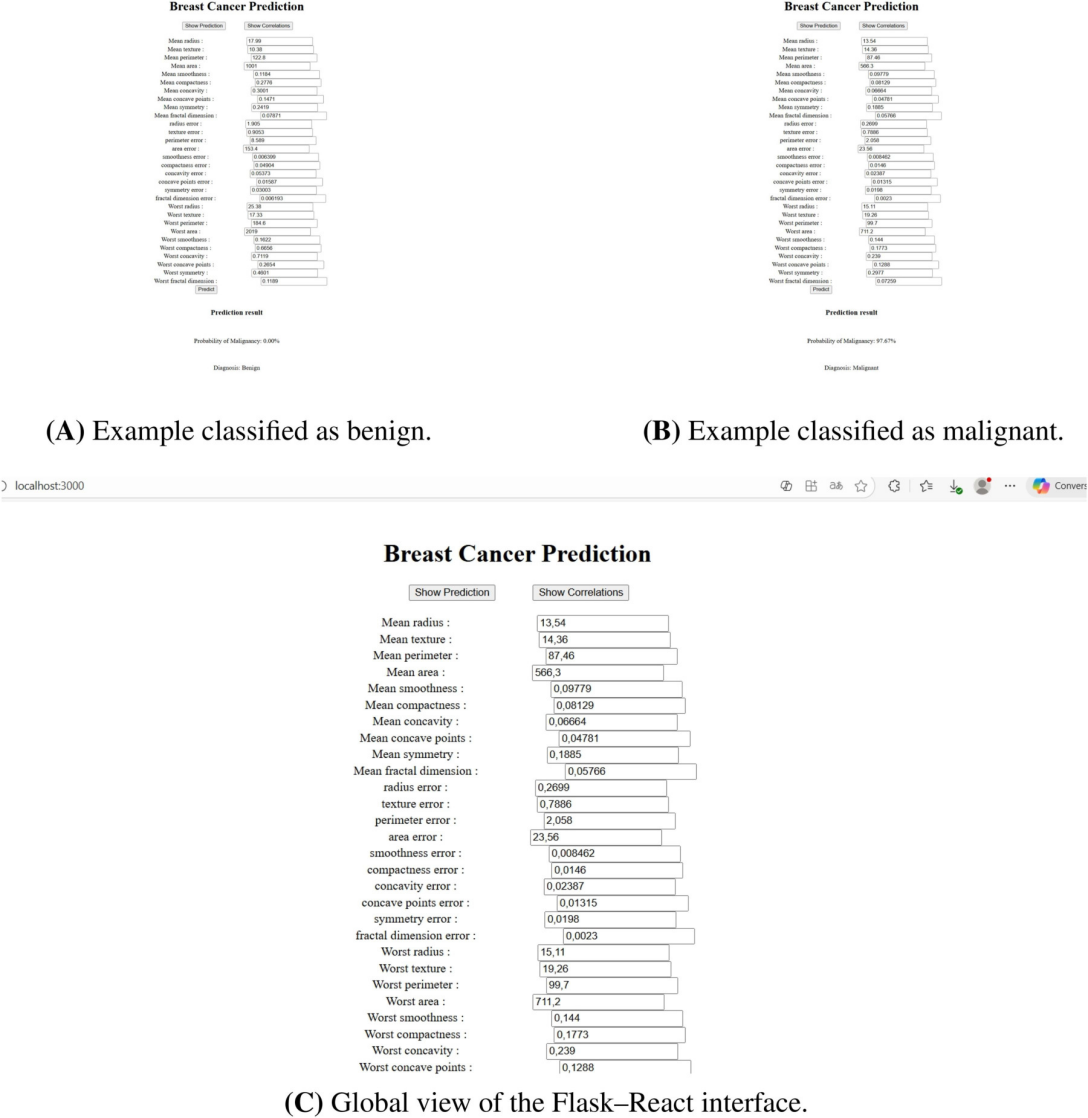
Flask–React web interface for LR–based tumour classification. **(A)** Prediction interface for a case classified as benign. **(B)** Prediction interface for a case classified as malignant. **(C)** Global view of the platform. **(A)** Example classified as benign. **(B)** Example classified as malignant. **(C)** Global view of the Flask–React interface.

Figure 11 summarizes the Flask–React prediction interface for tumour classification: two example cases (benign and malignant) and a global overview of the platform.

**Figure 12:**
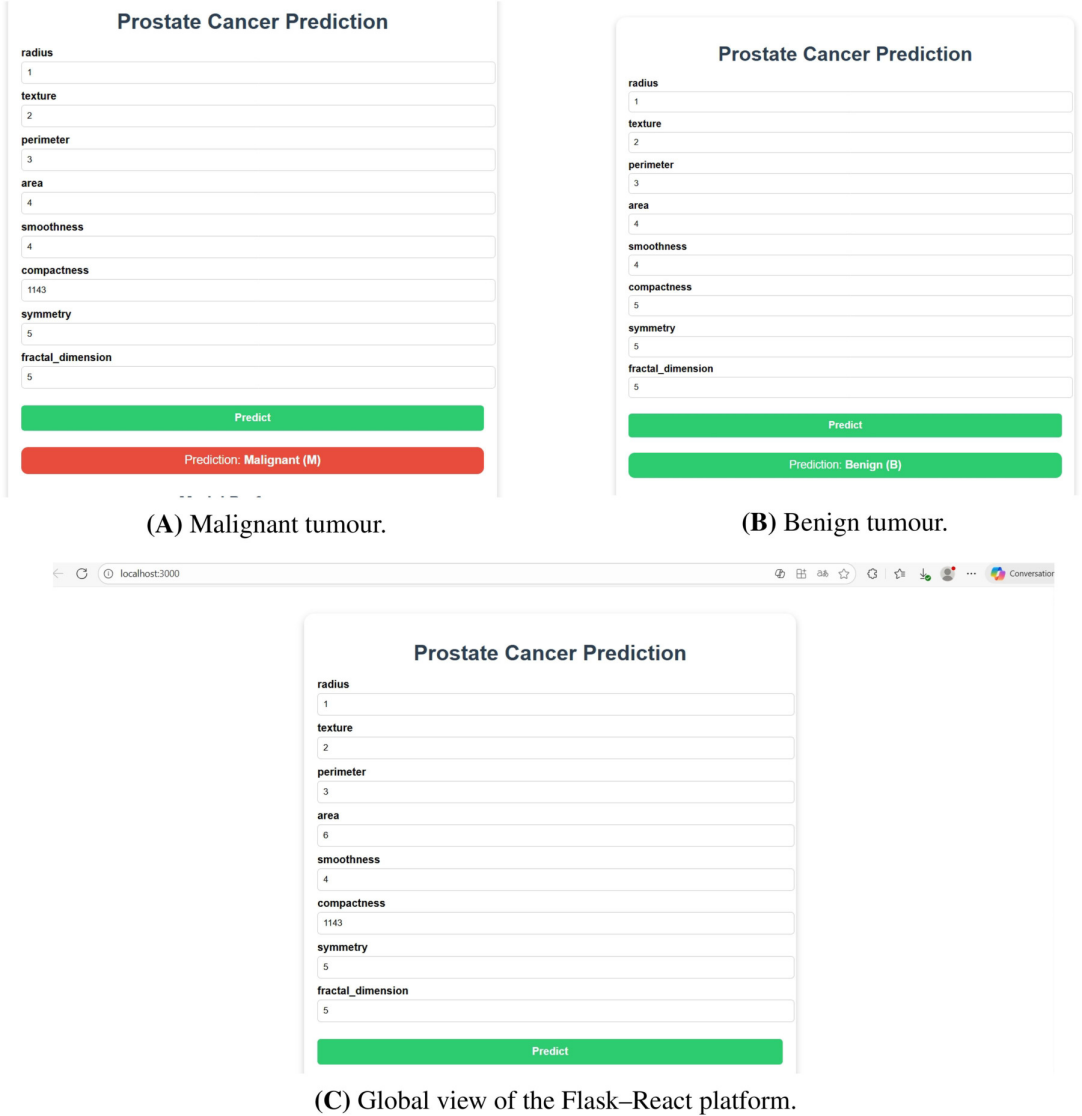
Flask–React web interface for LR-based prostate tumour classification. **(A)** Example prediction interface for a malignant case. **(B)** Example prediction interface for a benign case. **(C)** Global view of the platform. **(A)** Malignant tumour. **(B)** Benign tumour. **(C)** Global view of the Flask–React platform.

Figure 12 summarizes the Flask–React prediction interface for prostate tumour classification.

## 5 Discussions

### 5.1 Discussions and limitations

The experiments show that ridge-regularized logistic regression provides a stable and interpretable framework for binary tumour classification on both datasets. For the Breast Cancer Wisconsin dataset, the cross-validation-selected regularization parameter was λ=1.0, whereas for the Prostate Cancer dataset it was λ=10.0. In both cases, the non-regularized model λ=0 was retained as an ablation baseline. This design makes it possible to distinguish between two effects: the predictive contribution of ridge regularization and the numerical behaviour of the optimization algorithms.

The Breast Cancer results indicate that all five optimizers reach nearly identical predictive performance at the selected value of λ, with high accuracy, AUC, sensitivity, specificity, and F1-score. The main difference between the optimizers is therefore not the final classification outcome, but their computational behaviour. Newton–Raphson and Conjugate Gradient reach the strict convergence criterion rapidly, whereas Gradient Descent requires many more iterations. BFGS and L-BFGS also return successful optimization statuses, although their final gradient norms may remain slightly above the strict threshold used in this study. This distinction between predictive equivalence and numerical efficiency is central to the proposed optimization analysis. This observation is consistent with previous studies on logistic-regression optimization, where Newton-type, trust-region, and quasi-Newton methods are often reported to be more efficient than plain steepest-descent approaches on smooth convex objectives (60–63).

The Prostate Cancer experiment leads to a similar conclusion, but with stronger evidence of the stabilizing effect of ridge regularization. Compared with the non-regularized case, the selected value λ=10.0 improves the clinical metrics and reduces the instability of the probabilistic predictions. The resulting accuracy, sensitivity, specificity, F1-score, AUC, and log-loss show that regularization is particularly useful on the smaller Prostate dataset, where the risk of unstable estimation is higher. The wider bootstrap confidence intervals observed for Prostate should therefore be interpreted in light of the limited sample size.

The Flask–React platform should be interpreted as a proof-of-concept deployment prototype rather than as a clinically validated diagnostic system. The optimization experiments were conducted using the custom logistic-regression objective, gradient, Hessian, and ridge-regularized training pipeline, whereas the Flask prototype uses the standard scikit-learn logistic-regression implementation for operational stability within the web interface. This distinction is important: the web platform demonstrates the feasibility of exposing an interpretable tumour-classification model through an interactive interface, but it does not replace prospective clinical validation.

Since the primary outcomes considered in this work are binary and the relationship between the features and the target variable is assumed to be linear, we adopt logistic regression as the primary modeling approach. Although methods such as XGBoost or LightGBM could also be considered, these algorithms are generally more appropriate when complex nonlinear interactions among features are expected. Furthermore, because the simulated datasets do not contain substantial noise, we are less motivated to employ tree-based procedures such as random forests. Methods such as support vector machines and neural networks are often advantageous in high-dimensional settings or with very large datasets; however, they are typically less interpretable than logistic regression, which is an important consideration in the present study.Beyond its suitability for binary outcomes, logistic regression is particularly appropriate for the present study because its non-regularized and ridge-regularized formulations lead to smooth convex optimization problems. This makes it possible to compare GD, Newton–Raphson, CG, BFGS, and L-BFGS under the same objective function, gradient structure, stopping criterion, and convergence diagnostics. In contrast, models such as Random Forest, XGBoost, SVM, LightGBM, or neural networks rely on different hypothesis classes and optimization mechanisms. Some are ensemble-based, some involve margin-based objectives, and others lead to non-convex training problems. Therefore, including these models would shift the purpose of the study from a controlled optimization analysis of logistic regression toward a broader model-selection benchmark. Such an extended comparison is valuable, but it requires model-specific hyperparameter tuning, calibration analysis, and interpretability assessment, and is therefore left for future work.

Several limitations remain. First, the study uses two relatively small tabular datasets, especially the Prostate Cancer dataset with only 100 observations. Second, no external multi-centre clinical validation was performed. Third, the available datasets do not contain sufficient demographic or institutional variables to support a full fairness analysis. Fourth, the present work focuses on ridge regularization because it preserves a smooth differentiable objective, which is suitable for comparing GD, Newton–Raphson, CG, BFGS, and L-BFGS under a unified optimization framework. L1-regularized logistic regression would introduce a non-smooth objective and would require different optimization methods, such as proximal or coordinate-descent approaches. Finally, the Flask–React interface has not yet undergone formal usability testing with clinical users.

## 6 Conclusion

This study presented a unified and reproducible framework for analysing logistic regression in binary tumour classification. The work focused on the mathematical and computational behaviour of logistic regression under non-regularized and ridge-regularized formulations, using Gradient Descent, Newton–Raphson, Conjugate Gradient, BFGS, and L-BFGS under a common objective, preprocessing protocol, and evaluation setting.

The experiments show that ridge regularization improves numerical stability and probabilistic behaviour, particularly on the smaller Prostate Cancer dataset. At the selected regularization levels, the five optimizers often produce comparable predictive performance, but they differ clearly in convergence speed, runtime, number of iterations, and strict convergence status. Thus, the main distinction between the optimizers is not only their final test accuracy, but their computational efficiency and stability under the same logistic-regression objective.

Clinically meaningful metrics, including sensitivity, specificity, precision, F1-score, ROC-AUC, log-loss, confusion matrices, and bootstrap confidence intervals, were incorporated to provide a more reliable assessment of the models. The additional external benchmark against standard machine-learning classifiers further shows that ridge-regularized logistic regression remains competitive while preserving coefficient-level interpretability and a clear optimization structure.

The Flask–React interface illustrates how an interpretable logistic-regression classifier can be exposed through an interactive web prototype. However, this platform should be interpreted as a proof-of-concept deployment component rather than as a clinically validated diagnostic system. Overall, the proposed framework contributes a transparent, reproducible, and clinically oriented optimization analysis of logistic regression for tumour classification, while leaving broader clinical validation, fairness analysis, and extended model benchmarking for future work.

## Data Availability

Publicly available datasets were analyzed in this study. This data can be found here: The data used in this study are publicly available. The Breast Cancer Wisconsin (Diagnostic) dataset can be accessed from the UCI Machine Learning Repository: https://archive.ics.uci.edu/ml/datasets/breast+cancer+wisconsin+(diagnostic). The prostate cancer dataset is available in the public GitHub repository “PROSTATE-CANCER-DETECTION”: https://github.com/SAZZZO99/PROSTATE-CANCER-DETECTION/tree/master.

## Code availability

The complete source code of the Flask–React web applications developed in this work is openly available online.

- Prostate cancer logistic regression platform (Flask–React): https://github.com/houssa677/flask-react-prostate-cancer
- Breast cancer logistic regression platform (Flask–React): https://github.com/houssa677/flask-react-breast-cancer

Each repository contains the Flask backend, the React frontend.
